# Cell-free DNA 5-hydroxymethylcytosine is an emerging marker of acute myeloid leukemia

**DOI:** 10.1038/s41598-022-16685-3

**Published:** 2022-07-20

**Authors:** Jianming Shao, Sihan Wang, Diana West-Szymanski, Jason Karpus, Shilpan Shah, Siddhartha Ganguly, Janice Smith, Youli Zu, Chuan He, Zejuan Li

**Affiliations:** 1 Department of Pathology and Genomic Medicine, Houston Methodist Hospital, Houston, TX USA; 2 Department of Medicine, The University of Chicago, Chicago, IL USA; 3 Department of Chemistry, Department of Biochemistry and Molecular Biology, Institute for Biophysical Dynamics, The University of Chicago, Chicago, IL USA; 4 Cancer Center, Houston Methodist Hospital, Houston, TX USA; 5 Weill Cornell Medical College, New York, NY USA; 6 Molecular and Human Genetics, Baylor College of Medicine, Houston, TX USA; 7 Howard Hughes Medical Institute, The University of Chicago, Chicago, IL USA; 8 Houston Methodist Research Institute, Houston, TX USA

**Keywords:** Diagnostic markers, Prognostic markers, Haematological cancer

## Abstract

Aberrant changes in 5-hydroxymethylcytosine (5hmC) are a unique epigenetic feature in many cancers including acute myeloid leukemia (AML). However, genome-wide analysis of 5hmC in plasma cell-free DNA (cfDNA) remains unexploited in AML patients. We used a highly sensitive and robust nano-5hmC-Seal technology and profiled genome-wide 5hmC distribution in 239 plasma cfDNA samples from 103 AML patients and 81 non-cancer controls. We developed a 5hmC diagnostic model that precisely differentiates AML patients from controls with high sensitivity and specificity. We also developed a 5hmC prognostic model that accurately predicts prognosis in AML patients. High weighted prognostic scores (wp-scores) in AML patients were significantly associated with adverse overall survival (OS) in both training (*P* = 3.31e−05) and validation (*P* = 0.000464) sets. The wp-score was also significantly associated with genetic risk stratification and displayed dynamic changes with varied disease burden. Moreover, we found that high wp-scores in a single gene, *BMS1* and *GEMIN5* predicted OS in AML patients in both the training set (*P* = 0.023 and 0.031, respectively) and validation set (*P* = 9.66e−05 and 0.011, respectively). Lastly, our study demonstrated the genome-wide landscape of DNA hydroxymethylation in AML and revealed critical genes and pathways related to AML diagnosis and prognosis. Our data reveal plasma cfDNA 5hmC signatures as sensitive and accurate markers for AML diagnosis and prognosis. Plasma cfDNA 5hmC analysis will be an effective and minimally invasive tool for AML management.

## Introduction

Acute myeloid leukemia (AML) is a devastating hematological malignancy characterized by epigenetic aberrations^1^. Mutations in epigenetic regulators are significantly overrepresented in pre-leukemic states and present in the majority of AML patients^2^. Although molecular profiling by next-generation sequencing (NGS) has been widely used for AML risk classification, its application for early diagnosis is limited^3^. Many prognostic factors, including age, cytogenetic and molecular abnormalities, white blood cell count, serum lactate dehydrogenase, and the presence of antecedent hematologic disorders are used to select appropriate therapy for AML patients^4,5^. However, the 5-year survival rate for AML patients 20 years and older is only 25% (Cancer.Net). Therefore, additional markers that provide early detection and accurate prognosis prediction to guide therapy are urgently needed.

Recent evidence indicates that 5-hydroxymethylcytosine (5hmC) correlates with tumorigenesis and gene expression^6^. 5hmC is the first oxidative product in the demethylation of 5-methylcytosine (5mC), which is catalyzed by ten-eleven-translocation proteins (TET1, TET2, and TET3)^7^. Unlike 5mC, which displays relatively constant levels in genomes across tissues, 5hmC is an active DNA demethylation mark enriched mostly in active genomic loci. 5hmC can be highly tissue-specific and stable but may also function as a transient intermediate^8^. Global loss of 5hmC has been observed in AML and many other cancers^9–12^ and is associated with *TET2*, *IDH1*, and *IDH2* mutations in some^11–13^ but not all^9,14–16^ patients. Recently, a selective chemical labeling method, termed nano-hmC-Seal technology, enables precise mapping of genome-wide 5hmC distributions with input DNA as low as 1–2 ng^17^. In conjunction with NGS, nano-hmC-Seal has been applied to plasma cell-free DNA (cfDNA) and revealed 5hmC as a promising diagnostic and prognostic marker in many cancers, including colorectal, gastric, thyroid, lung, liver, esophageal, pancreatic cancers, and lymphoma^18–22^. Whole-genome 5hmC maps in 19 human tissues have recently been reported, demonstrating tissue-specific 5hmC marks on tissue-specific gene bodies and enhancers^23^. However, genome-wide profiling of 5hmC in the plasma cfDNA of AML patients has not yet been reported.

cfDNA contains DNA fragments originating from normal and tumor cells undergoing apoptosis, necrosis, or active secretion^24,25^. As AML originates in the bone marrow (BM) and extends to blood, most molecular analyses require blood and BM. However, many studies on hematological malignancies found that some clinically significant mutations were present in cfDNA but not BM or blood, though most mutations could be detected in all three sources^26–32^. A DNA methylation study in patients with myelodysplastic syndromes (MDS) showed earlier detection with cfDNA relative to BM^33^. Serial analysis of cfDNA has also demonstrated the utility of monitoring clonal dynamics and therapeutic response in chronic lymphocytic leukemia and multiple myeloma^34,35^. Taken together, these results indicate that cfDNA is an equivalent or superior molecular diagnostic tool that contains more comprehensive tumor information than blood or BM biopsy samples. BM biopsy is invasive and samples may not capture all of the malignant clones and surrounding microenvironment involved in the disease.

To investigate the role of 5hmC in AML, we collected 239 blood samples from 103 AML patients and 81 non-cancer controls and used the highly sensitive and specific nano-hmC-Seal method combined with NGS (nano-hmC-Seal-Seq) to profile the genome-wide distribution of 5hmC from plasma cfDNA. We evaluated the association of 5hmC with disease status, genetic characteristics, and survival of AML patients, and developed 5hmC models for diagnosis and prognosis.

## Results

### Genome-wide 5hmC profiling in plasma cfDNA

Normalized total 5hmC peak numbers for each sample were comparable in AML patients and controls, though we observed a wide range in AML samples (Supplementary Fig. 1a). 5hmC is enriched in CpG islands, promoters, untranslated regions (UTRs), exons, introns, transcription termination sites, microRNAs, and non-coding RNAs but not in intergenic regions in all samples (Supplementary Fig. 1b). We confirmed this finding in a chromHMM analysis of regulatory elements, which revealed that 5hmC is highly enriched in flanking regions of transcription start sites (TSS) and enhancers (Supplementary Fig. 1c). These findings are consistent with previous reports in tissues^23^ and cfDNA from solid tumors, including lung, pancreatic, and colorectal cancers^8^.

Compared with non-cancer controls, AML samples demonstrated a significant difference in 5hmC enrichment in genomic regions associated with active transcription. 5hmC levels in promoters, 5′UTRs, TSS flanking regions, and enhancers were significantly higher in AML than control samples (Supplementary Fig. 1b–d). 5hmC in AML was also highly enriched in genomic regions associated with active histone marks, such as H3K4me1, H3K4me3, H3K27ac, and H3K36me3, but not in genomic regions associated with repressive histone marks, such as H3K9me3 and H3K27me3 (Supplementary Fig. 1e).

### Differentially hydroxymethylated genes in AML

**Figure 1:**
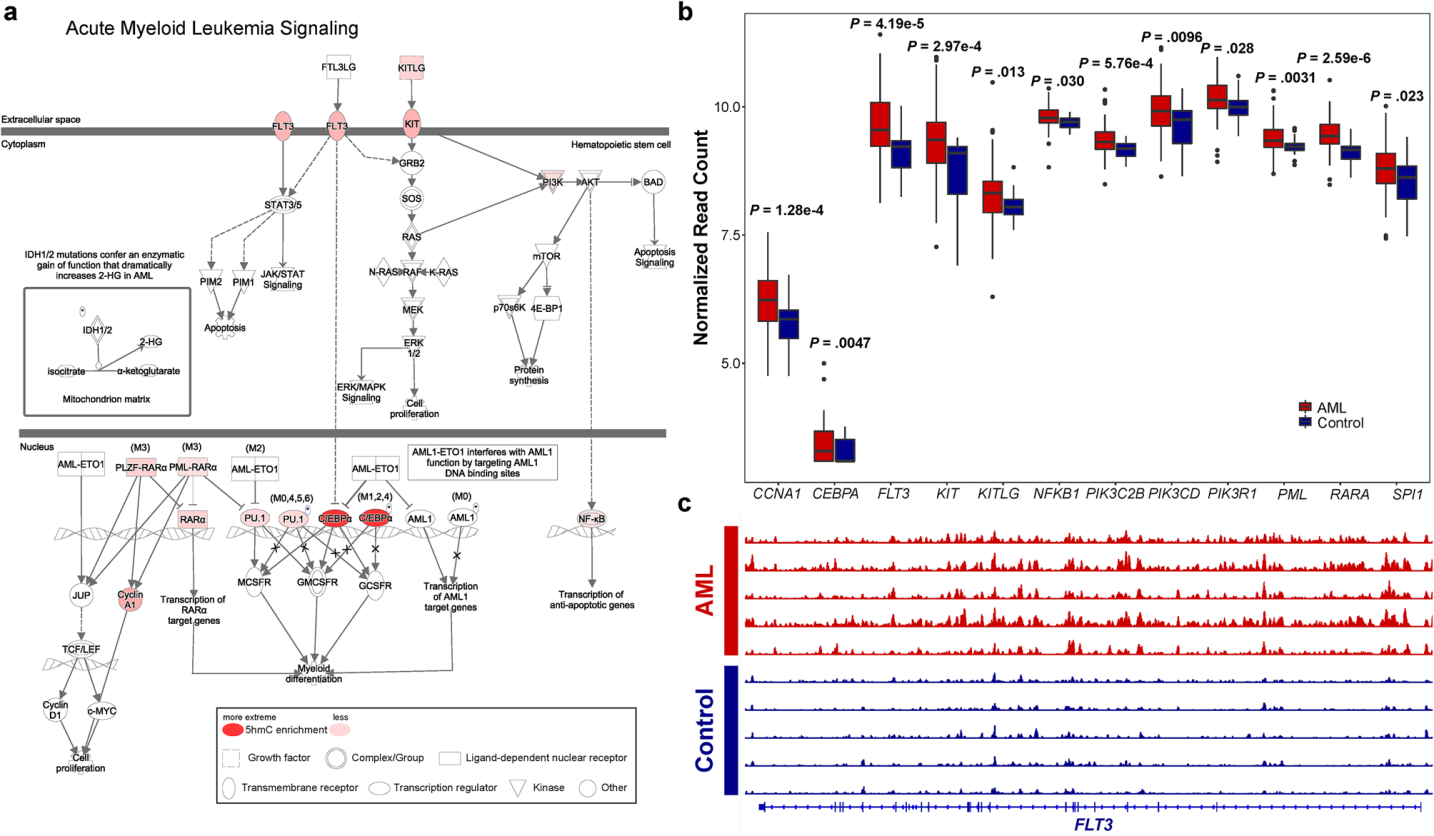
Differentially hydroxymethylated genes (DhMGs) involved in AML signaling pathways. (**a**) AML signaling pathways generated by IPA. (**b**) 5hmC levels in DhMGs associated with AML signaling. The DhMGs in AML signaling pathways are all enriched with 5hmC compared to controls (FDR < 0.01). Center line represents median, bounds of box represent 25th and 75th percentiles, and whiskers are Tukey whiskers. (**c**) Genome browser view of 5hmC distribution of *FLT3* in AML and control samples. Peak height represents 5hmC enrichment.

Compared to controls, we identified 2552 differentially hydroxymethylated genes (DhMGs) with increased 5hmC levels and 1678 DhMGs with decreased 5hmC levels in AML patient samples with overt leukemia at registration time (FDR < 0.01, Table S1. Unsupervised hierarchical clustering analysis using the 100 most significant DhMGs revealed an apparent separation between AML and control samples (Supplementary Fig. 2a). As 5hmC is an active DNA demethylation mark^8^, increased 5hmC modification of a gene may lead to gene upregulation. We correlated genome-wide 5hmC profiling with AML gene expression data generated from The Cancer Genome Atlas dataset and found 1158 DhMGs (FDR < 0.05) with concordant 5hmC and RNA expression levels (i.e., 704 DhMGs with increased 5hmC enrichment and RNA overexpression) (FDR < 0.05) and 454 DhMGs with reduced 5hmC levels and RNA downregulation. Among the concordant DhMGs, those with increased 5hmC levels were significantly enriched in signaling pathways and function categories related to cell proliferation, apoptosis, and immune regulation (Supplementary Fig. 2b, Supplementary Fig. 3a, and Table S2). In contrast, DhMGs with decreased 5hmC levels were associated with fewer signaling pathways and disease and function categories (Supplementary Fig. 2c, Supplementary Fig. 3b, and Table S2). Twelve DhMGs were involved in AML signaling pathway and all showed significant 5hmC enrichment in AML samples compared to controls (FDR < 0.01, Fig. 1a,b). Genes related to DNA methylation, demethylation, and chromatin modifications, such as *DNMT1*, *DNMT3A*, *DNMT3B*, *TET1*, *TET2*, *IDH1*, *IDH2*, *ASXL1*, and *EZH2*, did not show significant differences in 5hmC levels between AML and control samples (Supplementary Fig. 4). The normalized read tracks of DhMGs are illustrated in AML and control samples using *FLT3* as an example (Fig. 1c). These findings demonstrate the critical role of DNA hydroxymethylation in AML and identify relevant genes and pathways for further investigation of the molecular mechanisms underlying AML.

### Classification of AML by a 5hmC diagnostic model

**Figure 2:**
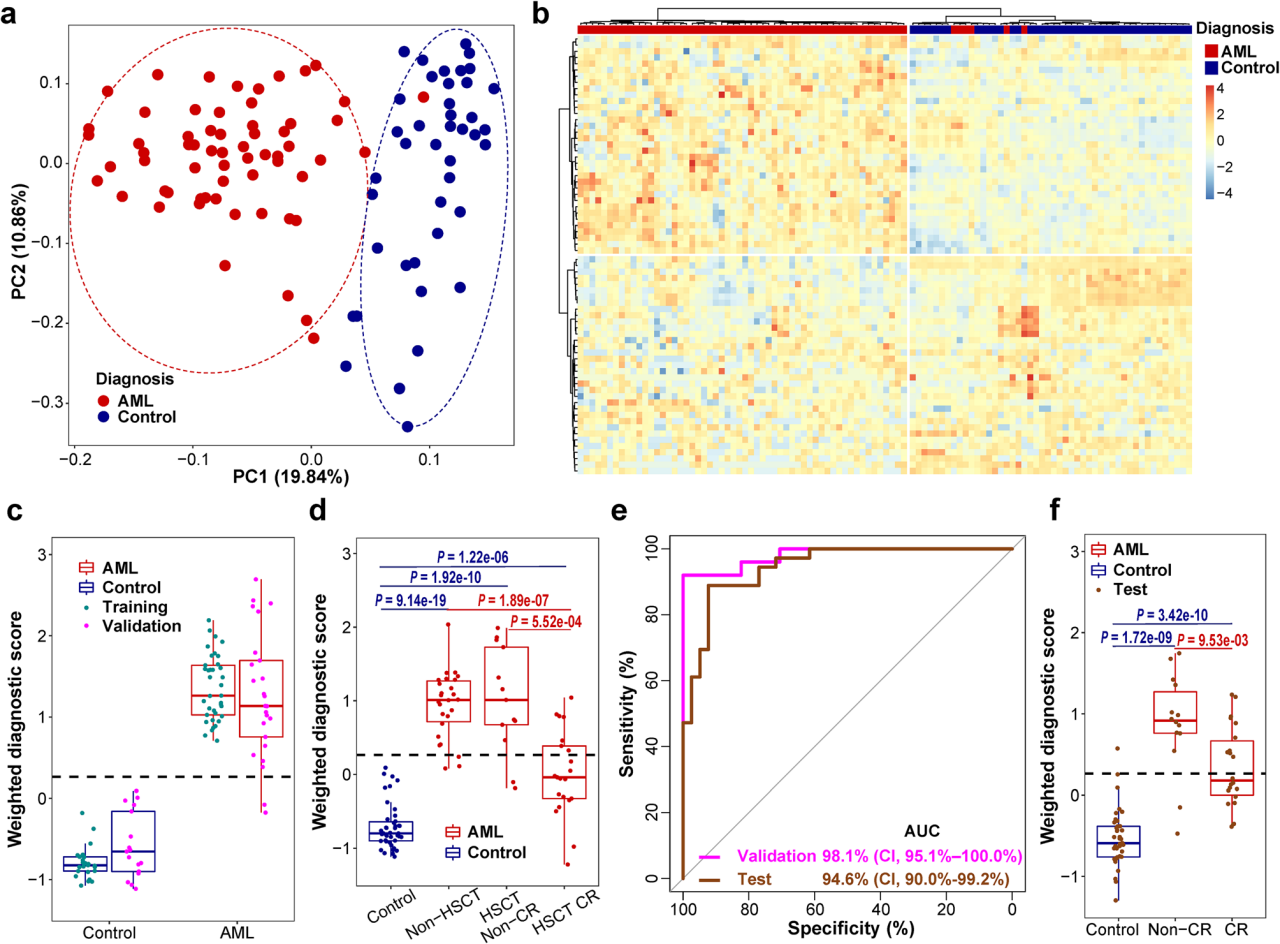
A 5hmC signature differentiates AML patients from controls. (**a**) Principal component analysis (PCA) in AML patient samples at registration time points and controls using normalized read counts from the 5hmC signature of 70 genes. (**b**) Unsupervised hierarchical clustering of the 5hmC signature of 70 genes in AML patient samples at registration time points and controls. (**c**) Boxplot of weighted-diagnostic scores (wd-scores) in controls and AML samples in both training (n = 62) and validation sets (n = 42). Black dashed line represents a cutoff score of 0.263 developed from the training set. *P* < 0.001, comparison of any of the AML groups to the control group in both training and validation sets. Center line represents median, bounds of box represent 25th and 75th percentiles, and whiskers are Tukey whiskers. (**d**) Boxplot of wd-scores in controls and other AML samples that were not included in the training, validation, and test sets. Non-HSCT, did not receive hematopoietic stem cell transplantation (n = 26); HSCT Non-CR, received hematopoietic stem cell transplantation and not in complete remission (n = 13); HSCT CR, received hematopoietic stem cell transplantation and in complete remission (n = 21). Center line represents median, bounds of box represent 25th and 75th percentiles, and whiskers are Tukey whiskers. (**e**) Receiver Operating Characteristics (ROC) analysis of wd-score calculated from the 5hmC signature of 70 genes in validation and test sets. *AUC* area under curve. *CI* 95% confidence interval. (**f**) Boxplot of wd-scores in additional AML samples and controls in the test set. Controls, n = 39. *Non-CR* did not achieve complete remission, n = 14. *CR* complete remission, n = 22. Center line represents median, bounds of box represent 25th and 75th percentiles, and whiskers are Tukey whiskers.

To develop a 5hmC diagnostic model capable of differentiating AML samples from controls, we first analyzed samples collected before 2020. AML patient samples with overt leukemia at registration time and control samples were randomly split into a training set (AML n = 37; control n = 25) and validation set (AML n = 25; control n = 17) at a 6 to 4 ratio (Supplementary Fig. 5, Table S3). By comparing 5hmC levels in AML samples and controls in the training set, 3048 candidate genes showed different 5hmC enrichment (*P* < 0.01). In these candidates, we further discovered a signature of 70 genes (Table S4) that separated AML samples at registration time (Fig. 2a,b) or all AML samples (Supplementary Fig. 6a,b) from controls in principal component and unsupervised hierarchical clustering analyses. We developed a 5hmC diagnostic model and calculated a weighted diagnostic score (wd-score) representing the 5hmC level of the 70 genes for each sample. AML samples had significantly higher wd-scores compared to controls in both training and validation sets (*P* < 0.001, Fig. 2c). Compared to controls, wd-scores were also significantly higher in AML samples collected at other time points in all treatment groups (Fig. 2d). At a wd-score cutoff of 0.263, the diagnostic model achieved an area under curve (AUC) of 98.1% [95% confidence interval (CI) 95.1–100%] in the validation set (Fig. 2e). Excluding the samples in the training set, with 100.0% specificity, the sensitivity of the diagnostic model was 92.0% in the validation set, 87.5% in AML patients who had not received hematopoietic stem cell transplant (HSCT), 87.7% in patients who had received HSCT not achieving complete remission (CR), and 33.3% in patients who had received HSCT in CR (Table 1).

**Table 1:** Prediction using the 5hmC diagnostic model in AML patients.

	AML validation set	Non-HSCT	HSCT non-CR	HSCT CR	Control validation set
Wd-score high	23	21	13	7	0
Wd-score low	2	3	2	14	17
Total	25	24	15	21	17
Sensitivity	92.0%	87.5%	87.7%	33.3%	–
Specificity	–	–	–	–	100%

*Non-HSCT* did not receive hematopoietic stem cell transplantation, *HSCT Non-CR* received hematopoietic stem cell transplantation and not in complete remission, *HSCT CR* received hematopoietic stem cell transplantation and in complete remission.

To further validate the diagnostic model, we performed genome-wide 5hmC profiling in a test set of 36 additional plasma cfDNA samples from 27 AML patients collected in 2020 and 2021 and 39 control samples (Table S3; Supplementary Fig. 5). We calculated a wd-score for each sample using the 5hmC diagnostic model. The wd-scores were significantly higher in AML samples than in controls (*P* = 1.72−e09 and 3.42e−10, respectively; Fig. 2f). The wd-scores in samples from patients with overt leukemia were also significantly higher compared to those in CR (*P* = 9.53−e03; Fig. 2f). In the test set, the AUC of the diagnostic model was 94.6% (CI 90.0–99.2%; Fig. 2e). With a specificity of 97.4%, the sensitivity of the diagnostic model was 85.7% in AML patients not achieving CR and 45.5% in patients in CR (Table S5). These results indicate that the 5hmC signature is a sensitive diagnostic marker for AML.

As mutations in *TET2*, *IDH1*, and *IDH2* may result in reduced global 5hmC production^36^, we evaluated whether these gene mutations impacted diagnostic scores. There was no significant difference in wd-scores between patients with and without a *TET2*, *IDH1*, or *IDH2* mutation, respectively (Supplementary Fig. 7a–c). This result suggests that the predictive ability of the 5hmC diagnostic model is independent of mutations in *TET2, IDH1*, and *IDH2*.

### A 5hmC model predicts prognosis of AML patients

**Figure 3:**
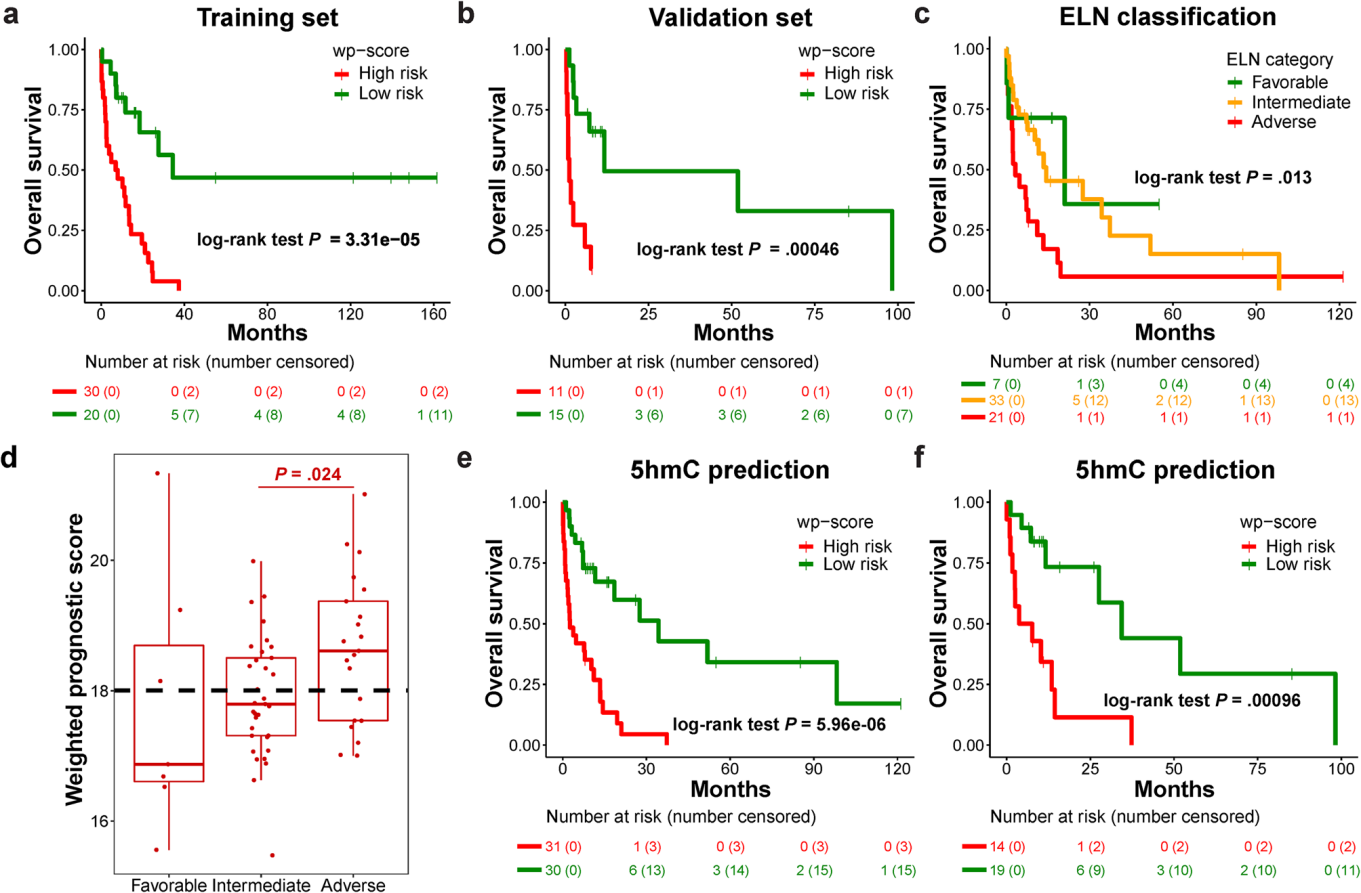
A 5hmC signature predicts overall survival in AML patients. (**a**) Kaplan–Meier analysis of overall survival of AML patients in training set based on wp-scores (n = 50). (**b**) Kaplan–Meier analysis of overall survival of AML patients in validation set based on wp-scores (n = 26). (**c**) Overall survival analysis of 61 AML patients with cytogenetic and/or molecular genetic information following the European LeukemiaNet (ELN) risk classification. (**d**) Association of wp-score with risk stratification of the 61 AML patients categorized using ELN classification. Favorable, n = 7; Intermediate, n = 33; Adverse, n = 21. Black dashed line represents a cutoff wp-score of 18.0 developed from training set. Center line represents median, bounds of box represent 25th and 75th percentiles, and whiskers are Tukey whiskers. (**e**) Overall survival analysis of the 61 AML patients with cytogenetic and/or molecular information available based on wp-scores. (**f**) Wp-score further stratifies patients in the intermediate group (n = 33) categorized by ELN recommendations.

To evaluate the association of 5hmC with overall survival (OS), we randomly split the AML samples collected before 2020 and at registration time points into a training set (n = 50) and validation set (n = 26; Supplementary Fig. 5). In the training set, through univariate analysis of the 4670 genes with differential 5hmC levels between AML and control samples, we identified 279 genes significantly associated with OS (*P* < 0.05). These genes were enriched in signaling pathways and disease and functional categories related to cancer, hematological diseases, hematopoiesis, and cell growth and proliferation (Supplementary Fig. 8a,b). Through further feature selection, we discovered a signature of three genes (*ODF3B*, *AC092691.1*, and *AC009035.1*) that was significantly associated with OS in the training set (Supplementary Fig. 9). A weighted prognostic score (wp-score) representing the 5hmC level of the three genes was generated for each sample. Wp-scores exceeding 18.0 were significantly associated with adverse OS of AML patients in both the training set (*P* = 3.31e−05, Fig. 3a) and validation set (*P* = 0.000464, Fig. 3b). Wp-scores were also significantly associated with OS in AML patients who received (*P* = 0.00719) or did not receive (*P* = 9.34e−05) HSCT (supplementary Fig. 10,b). Moreover, we demonstrated that high wp-scores were significantly associated with shorter event-free survival (EFS) (*P* = 0.0234; Supplementary Fig. 11). Wp-scores were also significantly higher in post-HSCT patients who later became relapsed relative to those that remained in CR (*P* = 6.19e−6; Supplementary Fig. 12). Multivariate Cox proportional hazards model analysis demonstrated that the wp-score prediction was independent of age and sex (Table S6).

Based on European LeukemiaNet (ELN) recommendations^5^, 61 AML patients with cytogenetic and/or molecular information available were classified into favorable, intermediate, and adverse groups (Fig. 3c). Wp-scores in the adverse groups were significantly higher than the intermediate group (*P* = 0.024) but not significantly different compared to the favorable group (*P* = 0.11; Fig. 3d), indicating that wp-score could provide stratification independent of ELN classification. Wp-scores not only predicted prognosis in all 61 patients (*P* = 5.96e−06; Fig. 3e) but also further refined those in the intermediate group into favorable and adverse subgroups (*P* = 0.00096; Fig. 3f). This result suggests potential clinical application of the 5hmC predictive model to further stratify patients in ELN risk classification in AML.

### The survival-associated 5hmC signature correlates with leukemia burden

**Figure 4:**
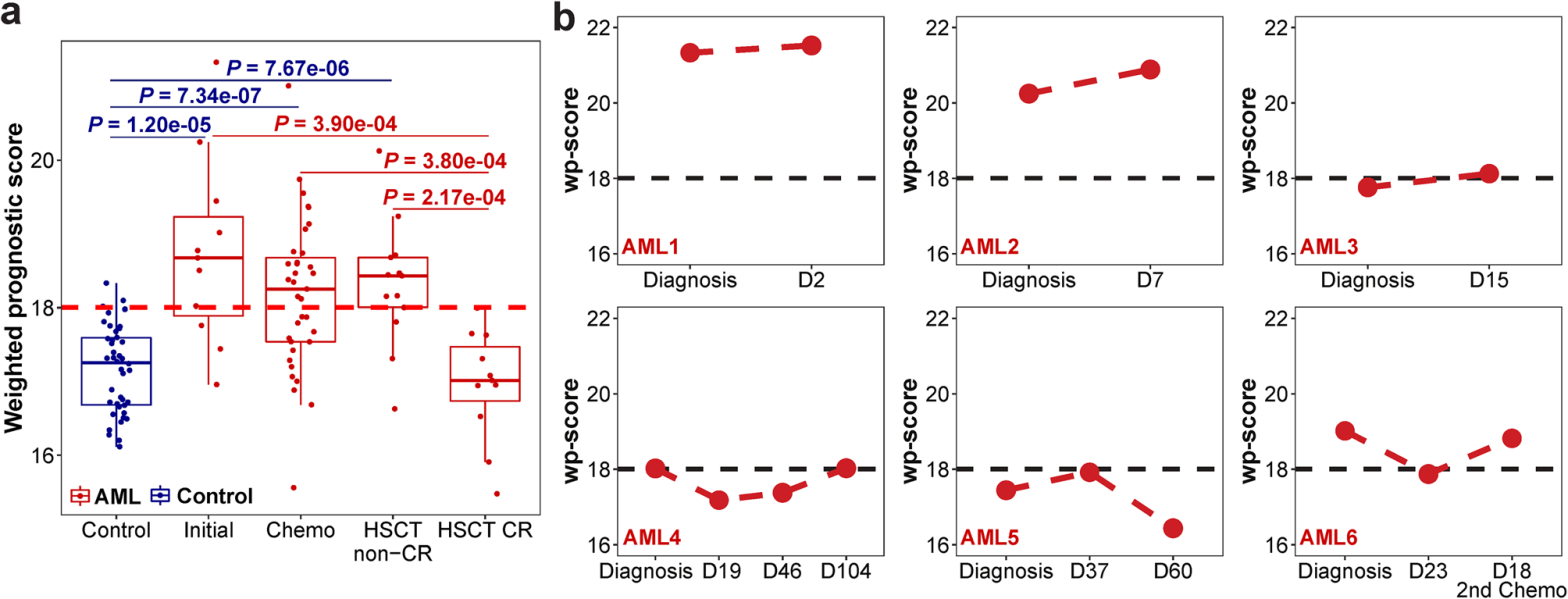
The survival-related 5hmC signature is associated with leukemia burden. (**a**) Boxplot of wp-scores in controls (n = 42) and patient samples at initial diagnosis (n = 11), with chemotherapy (n = 35), after receiving hematopoietic stem cell transplant not in complete remission (HSCT non-CR; n = 13) or HSCT in CR (HSCT CR; n = 11). Red dashed line represents a cutoff wp-score of 18.0 developed from training set. Center line represents median, bounds of box represent 25th and 75th percentiles, and whiskers are Tukey whiskers. (**b**) Plots of wp-scores in individual AML patients at initial diagnosis and after chemotherapy. Dx, days after initiation of chemotherapy. Black dashed line represents the cutoff wp-score.

The survival-related 5hmC signature of three genes was also significantly associated with disease burden. Compared to controls, wp-scores were significantly higher in patients who did not achieve CR (Fig. 4a). There was no significant difference between patients with CR and controls (*P* = 0.51; Fig. 4a). Wp-scores also showed dynamic changes during the course of chemotherapy in individual patients. In patients AML1, AML2, and AML3, wp-scores did not decrease in the first few days of chemotherapy, while in patients AML4, AML5, and AML6, wp-scores decreased after a longer period of treatment (at days 19, 60, and 23 of chemotherapy, respectively, Fig. 4b). Wp-scores eventually increased at later stages in patients AML4 and AML6 (Fig. 4b). In AML1 and AML2, wp-scores at initial diagnosis were higher than in other patients; further, they died of AML at day 3 and day 19 after diagnosis, respectively. The dynamic feature of 5hmC in plasma indicates that 5hmC could be a promising marker for minimal residue disease (MRD) monitoring in AML.

### Other survival predictive genes

**Figure 5:**
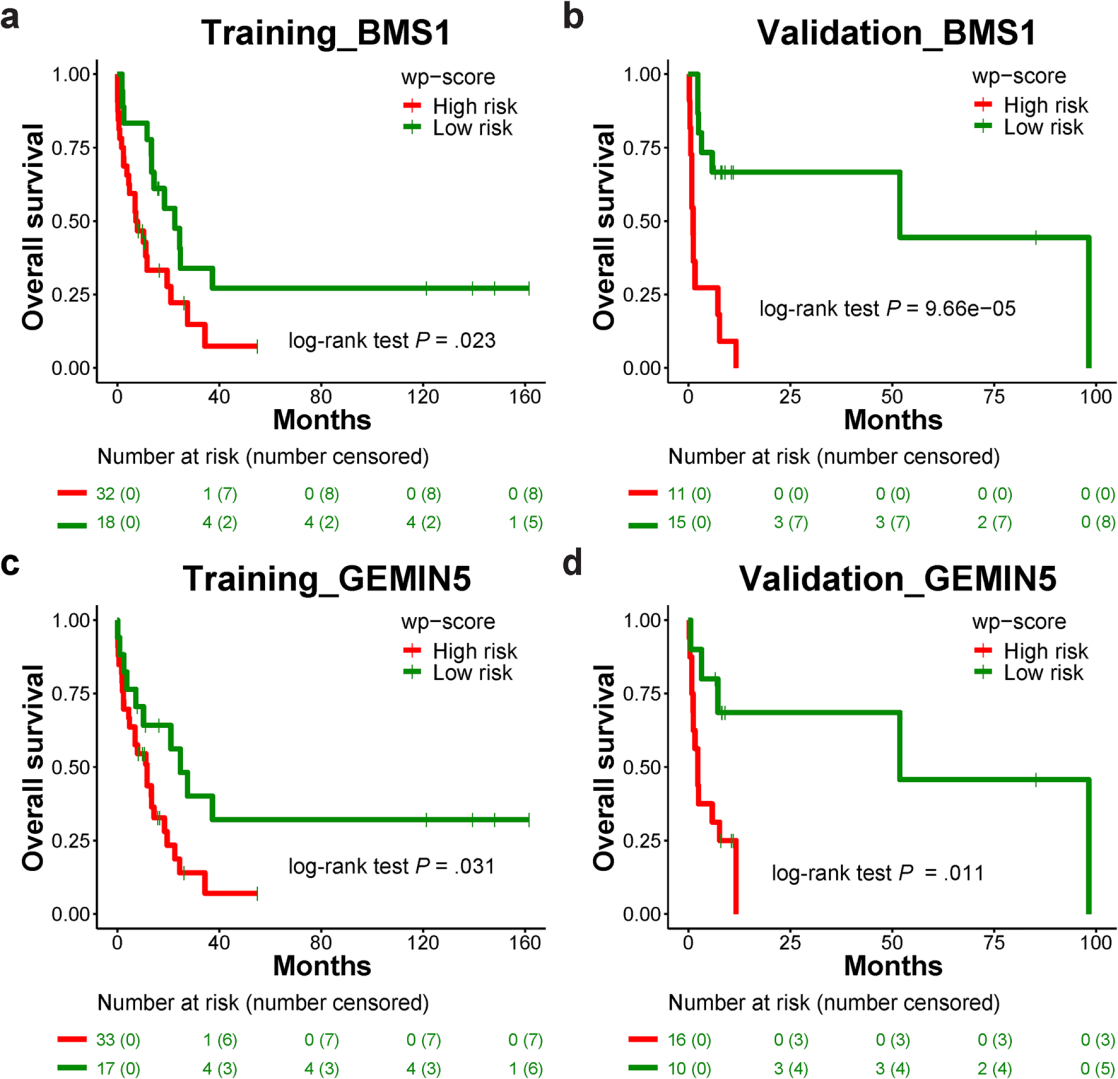
A 5hmC levels in a single gene for survival prediction. (**a**) Kaplan–Meier analysis of overall survival of AML patients in training set based on wp-scores of *BMS1* (n = 50). (**b**) Kaplan–Meier analysis of overall survival of AML patients in validation set based on wp-scores of *BMS1* (n = 26). (**c**) Kaplan–Meier analysis of overall survival of AML patients in training set based on wp-scores of *GEMIN5* (n = 50). (**d**) Kaplan–Meier analysis of overall survival of AML patients in validation set based on wp-scores of *GEMIN5* (n = 26).

Among 279 survival-related genes, we sought to identify the best single 5hmC gene marker that could predict prognosis. In the training set of AML patients (n = 50), we calculated a wp-score of each gene for each sample and determined a cutoff for each gene to classify the patients into a high or low-risk group. We then validated the performance of the wp-scores in the validation set (n = 26). The wp-scores of 157 genes significantly predicted OS in the training set (*P* < 0.05), but only 19 genes showed significant association with OS in the validation set (*P* < 0.05). Among the 19 genes, *BMS1* and *GEMIN5* were the top two predictors. Both genes showed significantly reduced 5hmC levels as well as decreased gene expression in AML samples compared to controls (Table S1). High wp-scores of *BMS1* and *GEMIN5* were significantly associated with adverse OS in AML patients in both training set (*P* = 0.023 and 0.031, respectively) and validation set (*P* = 9.66e−05 and 0.011, respectively; Fig. 5a–d).

## Discussion

We present a 5hmC diagnostic model in plasma cfDNA that accurately distinguishes AML samples from controls. Aberrant DNA methylation occurs early in neoplastic development and has been investigated as a diagnostic marker in AML^2,37,38^. DNA hypomethylating agents, such as 5-azacitidine and decitabine, are standard therapy for patients with AML and MDS^39^. Genome-wide profiling of 5hmC in plasma cfDNA showed a wide distribution of total 5hmC peak numbers, consistent with a previous report in AML^13^. The 5hmC model developed herein based on a selected group of genes can precisely reflect the presence of malignancies in AML patients. Compared with DNA methylation profiling, 5hmC analysis using the nano-hmC-Seal method requires less DNA, has lower sequencing costs, and higher sequencing quality (as no bisulfite treatment is involved). In contrast to whole blood analysis, 5hmC analysis may capture trace amounts of malignant cfDNA released by AML cells in BM before full-blown leukemia develops in peripheral blood. Plasma cfDNA 5hmC analysis also shows advantages over molecular profiling, which is confounded by clonal hematopoiesis of indeterminate potential^3^ for early diagnosis of AML. Along with other approaches, sensitive plasma cfDNA 5hmC analysis may facilitate accurate and early diagnosis of AML.

We also discovered a 5hmC signature in plasma cfDNA that was significantly associated with AML prognosis. AML is a heterogeneous malignancy with complex mechanisms and multiple biomarkers may be needed for disease prognosis. Currently, cytogenetic and molecular markers are the most important for risk stratification and treatment of AML patients^4,5^. Recent studies have demonstrated that epigenetic markers can be valuable for disease classification and clinical outcome prognosis in AML patients^13,40^. High global 5hmC levels are associated with inferior OS^13^. Our predictive model developed using a 5hmC gene signature or a single gene can significantly separate AML patients with a favorable prognosis from those with an adverse prognosis. The prediction was applicable to patients receiving chemotherapy alone or combined with HSCT. The wp-score of the three gene signatures in post-HSCT patients also predicted whether the patient would relapse at a later time. Based on the wp-score of the three gene signatures, patients in the intermediate-risk group classified by ELN recommendations were further separated into favorable and adverse groups. The 5hmC prediction was also independent of age and sex for AML prognosis. This result indicates potential for the 5hmC predictive model in a wide range of AML patients, working as either an independent prognosis predictor or in conjunction with other tests to facilitate the selection of treatment regimens for AML patients.

We observed plasma cfDNA 5hmC levels change dynamically with disease burden in AML patients as demonstrated by both the diagnostic and predictive models. The half-life of plasma is very short, within a few hours^41^. Using the sensitive 5hmC detection method, plasma cfDNA 5hmC may serve as a potential marker for real-time patient monitoring, MRD detection, and therapeutic response prediction.

Our study also revealed the genome-wide landscape of DNA hydroxymethylation in the plasma cfDNA of AML patients and found that 5hmC is enriched in functional regions in both AML and control samples. We discovered AML-specific gains or losses of hydroxymethylation. In AML patients, 5hmC enrichment was significantly higher in cis-regulatory elements compared to controls. Histone marks also regulate gene transcription^42^, and we found that 5hmC enrichment in AML is higher in chromatin marks associated with transcriptionally active genes, such as H3K4me1 and H3K27ac (in *cis*-regulatory enhancer elements), H3K4me3 (in promoters), and H3K36me3 (in gene bodies). However, 5hmC levels in chromatin marks associated with repressed genes (H3K9me3 and H3K27me3) were comparable between AML and control samples. Our findings provide additional evidence of the critical role of DNA demethylation in biological processes and diseases.

Identification of new AML biomarkers may also contribute to a better understanding of the molecular basis of the disease. In DhMGs involved in AML signaling, 5hmC was significantly enriched in transcription factors (*CEBPA*, *NFKB1*, *RARA*, and *SPI1*), tyrosine kinases (*FLT3* and *KIT*), the growth factor *KITLG*, phosphoinositide 3-kinase (PI3K) family members (*PIK3C2B*, *PIK3CD*, and *PIK3R1*) and the cell cycle regulator *CCNA1* in AML patients. Consistent with the 5hmC enrichment, these genes were upregulated in AML samples (Table S1) and play essential roles in the regulation of cell survival and proliferation, programmed cell death, cell migration, intracellular protein trafficking, hematopoiesis, and stem cell maintenance. The survival signature genes, *ODF3B*, *AC092691.1*, and *AC009035.1*, all showed significant enrichment of 5hmC in AML patients compared to controls (Table S1). However, the function of these genes has not been well studied in cancer yet. Moreover, we discovered that 5hmC levels of *BMS1* and *GEMIN5* predicted OS in AML patients. *BMS1* is a ribosomal biogenesis factor and highly conserved across species^43^. *GEMIN5* is an RNA-binding protein and is an essential component of spliceosomes^44^. The 5hmC levels and gene expression of *BMS1* and *GEMIN5* are both reduced in AML compared to controls, supporting the tumor suppressor nature of these genes. Our study provided new insights for the understanding of the underlying molecular mechanisms and revealed novel targets for future therapeutic interventions in AML.

There were some limitations in our study. The information for cytogenetics and molecular analyses was limited as many samples were collected before molecular genetic analyses were available. Moreover, as AML is a heterogeneous malignancy, 5hmC may have distinct role(s) in each subtype. Therefore, larger scale and prospective studies are warranted to confirm our findings.

In summary, we demonstrated that plasma cfDNA 5hmC is an emerging marker for AML diagnosis and prognosis. We also illustrated the important role of DNA hydroxymethylation in AML and discovered new targets to deepen our understanding of AML mechanisms. This study provides a proof of concept for the utility of plasma cfDNA 5hmC analysis as an accurate, sensitive, and minimally invasive diagnostic and prognostic tool in AML.

## Methods

### Patient cohort

We evaluated 158 plasma samples from 103 adult patients diagnosed with AML (Table S7) and 81 plasma samples from 81 non-cancer individuals. For the entire analyses, 122 plasma samples collected from 2005 to 2019 from 76 AML patients were used. For 5hmC diagnostic model validation, 36 plasma samples collected in 2020 and 2021 from 27 AML patients were used. The ages of non-cancer control patients ranged from 21 to 93 years (median 60 years) in 38 males and 43 females. The ages of AML patients ranged from 27 to 94 years (median 61 years). OS of AML patients before 2020 ranged from 0.1 to 162 months (median 8.8 months). OS was defined as time of registration to death. EFS of AML patients before 2020 ranged from 3.0 to 68.5 months (median 8.3 months). Patient ethnicities included Hispanic (16.5%) and non-Hispanic (83.5%). Thirty-seven of the AML patients had multiple blood collection time points (Table S8). Patients with acute promyelocytic leukemia were excluded from the study. One patient did not have treatment information on record. All other patients received chemotherapy (3 days of anthracycline and 7 days of cytarabine and/or hypomethylating agent). Among the patients sampled before 2020, 33 received HSCT. Twenty-one blood samples were collected at CR and post-HSCT. Blood samples were collected at initial diagnosis and during or after treatment and stored in the Biorepository Core at the Houston Methodist Hospital. Chromosomal karyotypes based on cytogenetic analysis and molecular alterations based on DNA fragment analysis or NGS were available from standard clinical care (Tables S7, S9 and S10). This study was approved by the institutional review board at Houston Methodist Hospital. All individuals signed informed consent forms to participate in the study.

### Sample preparation

Peripheral blood samples were collected in Vacutainer EDTA tubes (BD, Franklin Lakes, NJ) and stored at 4 °C before plasma isolation within 3 days. Plasma was separated from whole blood by centrifuge at 1350×*g* for 10 min at 4 °C and stored at − 80 °C. Plasma cfDNA was extracted using QIAamp Circulating Nucleic Acid Kit (QIAGEN, Germantown, MD) following manufacturer’s instructions. The cfDNA was quantified using the Qubit Fluorometer with dsDNA HS Assay Kit (Thermo Fisher Scientific, Waltham, MA) and Bioanalyzer 2100 with Agilent High Sensitivity Assay Kit (Agilent Technologies, Santa Clara, CA).

### 5hmC library construction and next-generation sequencing

5hmC library construction was performed as previously described^17^. Briefly, cfDNA was ligated with adaptors and was incubated with N3-UDP-azide-glucose and T4 Phage β-glucosyltransferase at 37 °C for 1 h. After purification, cfDNA was incubated with DBCO-PEG4-DBCO at 37 °C for 2 h. The captured DNA fragments were amplified and sequenced using the NextSeq 550 and NovaSeq 6000 instrument (Illumina, San Diego, CA). All experiments were performed in accordance with relevant guidelines and regulations.

### Identification of 5hmC enriched regions and differentially hydroxymethylated genes

We evaluated the quality of raw reads and trimmed adaptors and low-quality reads using Trimmomatic^45^. High-quality reads were mapped to the reference genome (GRCh37) using bowtie2 with the end-to-end mode^46^. Reads with mapping quality score ≥ 20, insert size < 600 bp, up to 1 ambiguous base, and < 3 mismatches were retained. We removed PCR duplicates using SAMtools^47^. To identify 5hmC-enriched regions, 5hmC peaks were called using MACS2 (false discovery rate, FDR < 0.01)^48^. Peaks within “blacklist regions” (downloaded from University of California Santa Cruz Genome Institute) and sex chromosomes were removed using bedtools^49^. We used HOMER annotatePeaks^50^ to analyze genomic feature enrichment. We obtained publicly available AML histone chromatin immunoprecipitation sequencing data and chromHMM segmentation data, an automated computational system for chromatin states, from the BLUEPRINT epigenome project (http://ftp.ebi.ac.uk/pub/databases/blueprint/releases/current_release/homo_sapiens/secondary_analysis/). Plotting and statistical tests were performed using R language version 3.6.3^51^.

To identify DhMGs, the number of high-quality reads mapped to gene body regions were counted without strand information using featureCounts software^52^. Raw read counts were normalized using variance-stabilizing transformation with DESeq2^53^. DhMGs (FDR < 0.01) were then identified using DESeq2 adjusted for sex and age. We performed unsupervised hierarchical clustering analysis using pheatmap (https://cran.r-project.org/web/packages/pheatmap/index.html). Metagene profiles of gene bodies and genomic regions associated with histone marks (AML data from BLUEPRINT project) were analyzed using ngsplot^54^ and deepTools^55^. Integrated genome viewer was used to visualize candidate genes of interest^56^. Gene enrichment analyses were performed with Ingenuity Pathway Analysis (IPA). AML differentially expressed genes were downloaded from the Gene Expression Profiling Interactive Analysis (GEPIA2) web server^57^.

### Development of a weighted diagnostic model for AML classification

To develop a plasma cfDNA 5hmC signature to classify AML from control samples, we used methods previously described^21^. Briefly, with a ratio of 6 to 4, we randomly split AML samples at initial collection time points (we excluded samples in CR) and control samples into a training set (37 AML and 25 control samples) and a validation set (25 AML and 17 control samples). Thirty-six AML samples collected in 2020 and 2021 and 39 controls were used as a test set. We performed univariate logistic regression analysis adjusted for age and sex from 37,468 genes and obtained 3048 informative markers with a cutoff *P* < 0.01 in the training set. To select the high confidence markers, the elastic net model was cross-validated for a grid of parameter values of α and λ (α range: 0.05–0.95 with 0.05 increment; λ range: 10^–5^–1 with logarithmically equal increments) using glmnet. This selection process was repeated 100 times and a list of 70 genes cross-validated in over 95% of sampling times was selected for the final diagnostic model. We then applied a multivariate logistic regression model to calculate the regression coefficient for each of the 70 genes, and calculated weighted diagnostic scores (wd-score) for each sample in the training, validation, and test sets, where wd-score $={\sum }_{\mathrm{k}=1}^{\mathrm{n}}\left({\upbeta }_{\mathrm{k}}\times {\mathrm{G}}_{\mathrm{k}}\right)$^22^. β_k_ is the coefficient from the multivariate logistic regression analysis for gene k, and G_k_ is the normalized 5hmC read counts of the kth marker gene. Principal component analysis (PCA) analysis was performed using the prcomp function of R. The AUC and 95% confidence interval were calculated to evaluate model performance using pROC. A cutoff score simultaneously maximized with sensitivity and specificity was determined using optimal.cutpoints in the training set^58^. Sensitivity and specificity were calculated based on the cutoff wd-score in the validation and test sets.

### Development of a weighted prognostic model for AML survival

We selected samples at registration time points and randomly split them into a training set (50 AML samples) and validation set (26 AML samples). We performed univariate analysis of DhMGs using Cox proportional hazards model with a cutoff of *P* < 0.05 and obtained 279 informative genes in the training set. Functional enrichment analysis of 279 genes was performed using IPA. We performed elastic net regularization on the multivariate analysis of selected genes using Cox proportional hazards model, adjusting for age and sex. We also performed feature selection using glmnet and cross-validated the elastic net model with a grid of parameter values for α (α range: 0.05–1 with 0.05 increment). This selection process was repeated 100 times. We identified ten genes by cross-validating in at least 95% iterations (Supplementary Fig. 13). We then performed multivariate analysis of the ten genes using Cox proportional hazards model and identified three genes (*ODF3B*, *AC092691.1*, and *AC009035.1*) as final predictive markers that significantly contributed to OS. We calculated each wp-score based on the 3 genes for each sample in both training and validation sets, where wp-score $={\sum }_{\mathrm{k}=1}^{\mathrm{n}}\left({\upbeta }_{\mathrm{k}}\times {\mathrm{G}}_{\mathrm{k}}\right)$^22^. β_k_ is the coefficient from the multivariate analysis using Cox proportional hazards model for gene k, and G_k_ is the normalized 5hmC read counts of the kth marker gene. The wp-score cutoff of 18.0 that simultaneously maximized sensitivity and specificity was determined in the training set using optimal.cutpoints. We then validated the performance of wp-score in the validation set. To evaluate whether 5hmC was an independent factor for AML survival, we performed multivariate Cox proportional hazards model analysis including age, sex, and wp-score.

To identify single gene markers for OS prediction, we used the same training (n = 50) and validation (n = 26) sets used for 5hmC survival prognostic model development. For the 279 survival-related genes, normalized 5hmC read counts of each gene were fitted with Cox proportional hazards model adjusting for age and sex. A wp-score was calculated for each gene in each sample with the equation wp-score = β × G^22^. β is the coefficient of the gene from Cox proportional hazards model and G is the normalized 5hmC read counts of the gene. A wp-score cutoff that simultaneously maximized sensitivity and specificity was determined using optimal.cutpoints.

### Statistical analyses

For identification of 5hmC-enriched regions, plotting and statistical tests were performed using R language version 3.6.3. To identify DhMGs, DESeq2 was used to compare 5hmC levels between AML and control samples (FDR < 0.01). For survival analysis, a Kaplan–Meier estimator was used in patients with high or low wp-scores. The log-rank test was used to evaluate statistical significance for the Cox proportional hazards models. For all other statistical tests, the Wilcoxon rank-sum test was used. A *P* value < 0.05 was considered significant.

### Ethics declarations

This study was approved by the institutional review board at Houston Methodist Hospital. The informed consent was waived.

## Supplementary Information

Supplementary Information 1.

Supplementary Table S1.

Supplementary Table S2.

Supplementary Table S10.

## Data Availability

The raw 5hmC sequencing data supporting the conclusions of this article are available in the National Center for Biotechnology Information (NCBI) Gene Expression Omnibus (GEO) database, accession number GSE163846 (https://www.ncbi.nlm.nih.gov/geo/query/acc.cgi?acc=GSE163846).
