# Supplementary material for: Cell-free DNA 5-hydroxymethylcytosine is an emerging marker of acute myeloid leukemia
Source: Sci Rep. 2022 Jul 20;12:12410. doi: 10.1038/s41598-022-16685-3 (PMC9300744; doi:10.1038/s41598-022-16685-3)
Supplement: Supplementary file 1 — Supplementary Information 1. [file 41598_2022_16685_MOESM1_ESM.docx]

**Supplementary Information**

**Cell-free DNA 5-hydroxymethylcytosine is an emerging marker of acute myeloid leukemia**

Jianming Shao, Sihan Wang, Diana West-Szymanski, Jason Karpus, Shilpan Shah, Siddhartha Ganguly, Janice Smith, Youli Zu, Chuan He, Zejuan Li

**Table S1.** List of differentially hydroxymethylated genes. See attachment.

**Table S2.** Differentially hydroxymethylated genes in top pathways of pathway enrichment analysis. See attachment.

**Table S3.** Characteristics of acute myeloid leukemia (AML) patients in the training, validation, and test sets.

**Table S4.** Diagnostic 5hmC signature of 70 DhMGs.

**Table S5.** Prediction using the 5hmC diagnostic model in the test set of AML patients.

**Table S6.** Multivariate Cox proportional hazards model analysis of age, sex, and wp-score in association with AML survival.

**Table S7.** Characteristics of AML cohort.

**Table S8.** Number of blood samples per AML patient in this study.

**Table S9.** Cytogenetics results of AML patients.

**Table S10.** Molecular results of AML patients. See attachment.

**Figure S1.** Genome-wide distribution of plasma cell-free DNA 5-hydroxymethylcytosine (5hmC) in AML and control samples.

**Figure S2.** Differentially hydroxymethylated genes (DhMGs).

**Figure S3.** Pathway enrichment analysis of DhMGs.

**Figure S4.** Boxplot of 5hmC enrichment in genes associated with epigenetic modifications.

**Figure S5.** Flow chart of study design of AML specimens for 5hmC diagnostic and predictive models.

**Figure S6.** A 5hmC signature of 70 genes differentiates all AML patients from controls.

**Figure S7.** 5hmC diagnostic signature in patients with or without a mutation in DNA demethylation related genes.

**Figure S8.** Pathway enrichment analysis of 279 survival-related DhMGs using Ingenuity Pathway Analysis.

**Figure S9.** A 5hmC signature of three genes significantly associated with overall survival.

**Figure S10.** Overall survival analysis of AML patients.

**Figure S11.** The survival-related 5hmC signature is associated with event-free survival in AML patients.

**Figure S12.** The survival-related 5hmC signature predicts leukemia burden in post-HSCT patients.

**Figure S13.** Candidate genes for final survival-related 5hmC signature.

**Table S3.** **Characteristics of AML patients in the training, validation and test set.**

|  | **Training set**  (No. of Patients) | **Validation set**  (No. of Patients) | **Test set**  (No. of Patients) |
| --- | --- | --- | --- |
| **Sex (male/female)** | 23/14 | 17/8 | 11/16 |
| **Age (range, median)** |  |  |  |
| All | 37 (30-78, 58 Yr) | 25 (27-88, 69 Yr) | 27 (31-94,63 Yr) |
| >=60 | 15 (60-78, 70 Yr) | 18 (60-88, 73 Yr) | 15 (61-94, 66 Yr) |
| <60 | 22 (30-59, 51 Yr) | 7 (27-55, 47 Yr) | 12 (31-59, 45 Yr) |
| **AML classification** |  |  |  |
| De novo | 33 | 23 | 21 |
| Secondary |  |  |  |
| from MDS | 2 | 1 | 4 |
| from myelofibrosis | 0 | 1 | 1 |
| from CML | 1 | 0 | 0 |
| from CMML | 0 | 0 | 1 |
| tAML | 1 | 0 | 0 |
| World Health Organization (WHO) |  |  |  |
| AML with recurrent genetic abnormalities |  |  |  |
| AML with t(8;21)(q22;q22.1) | 2 | 0 | 1 |
| AML with inv(16)(p13.1q22) or t(16;16)(p13.1;q22) | 1* | 2^ | 2 |
| AML with t(9;11)(p21.3;q23.3) | 1 | 0 | 1 |
| AML with *BCR-ABL1* | 1 | 2^ | 0 |
| AML with mutated *NPM1* | 3 | 2 | 4 |
| AML with myelodysplasia-related changes | 1 | 1 | 4 |
| Therapy-related myeloid neoplasms | 1* | 0 | 0 |
| AML, not otherwise specified (NOS) |  |  |  |
| AML without maturation | 1 | 0 | 0 |
| AML with maturation | 1 | 2 | 0 |
| Acute myelomonocytic leukemia | 2 | 1 | 0 |
| Acute monoblastic/monocytic leukemia | 2 | 1 | 0 |
| Pure erythroid leukemia | 1 | 1 | 0 |
| Unspecified | 21 | 14 | 15 |
| French-American-British (FAB) |  |  |  |
| M1 | 1 | 0 | 0 |
| M2 | 1 | 2 | 1 |
| M4 | 3 | 2 | 2 |
| M5 | 2 | 1 | 3 |
| M6 | 1 | 1 | 0 |
| Unspecified | 29 | 19 | 21 |
| **Treatment** |  |  |  |
| No treatment | 4 | 1 | 3 |
| Chemo | 32 | 24 | 24 |
| HSCT | 7 | 4 | 14 |
| No treatment info | 1 | 0 | 0 |
| **Cytogenetics** |  |  |  |
| Normal | 10 | 12 | 15 |
| Abnormal | 17 | 10 | 12 |
| Unknown | 10 | 3 | 0 |
| **Gene mutations**  (positive/total No. of patients tested) |  |  |  |
| *FLT3* | 3/5 | 3/7 | 5/27 |
| *NPM1* | 2/9 | 2/10 | 4/27 |
| Both *FLT3* and *NPM1* | 1/5 | 2/7 | 1/27 |
| *TET2* | 1/7 | 3/10 | 3/26 |
| *IDH1/2* | 1/8 | 2/11 | 7/26 |
| *DNMT3A* | 1/8 | 3/10 | 6/26 |
| *ASXL1* | 0/7 | 2/10 | 4/26 |
| *TP53* | 3/8 | 4/10 | 1/26 |

MDS, myelodysplastic syndrome; CML, chronic myelogenous leukemia; CMML, chronic myelomonocytic leukemia; tAML, therapy-related AML; HSCT, hematopoietic stem cell transplant; Yr, year old; * One patient with inv(16)(p13.1q22) also had therapy-related AML; ^ One patient with inv(16)(p13.1q22) also had *BCR-ABL1*.

**Table S4. Diagnostic 5hmC signature of 70 DhMGs.**

| **Gene Name** | **Position (GRCh37)** | **Coefficient** |
| --- | --- | --- |
| *KCTD11* | chr17:7255208-7258263 | 0.096445 |
| *LINC02552* | chr11:104316596-104480049 | 0.092322 |
| *ADGRL4* | chr1:79355449-79472415 | 0.091816 |
| *ATP10A* | chr15:25922420-26110319 | 0.089753 |
| *LINC02451* | chr12:43040385-43080503 | 0.082649 |
| *LINC00351* | chr13:85937736-86118705 | 0.076995 |
| *CA8* | chr8:61097971-61193959 | 0.076242 |
| *LINC00373* | chr13:88795122-88888337 | 0.075189 |
| *ADAM3A* | chr8:39308564-39380371 | 0.073122 |
| *AC090809.1* | chr8:36315501-36636714 | 0.072104 |
| *FAM83B* | chr6:54711569-54809897 | 0.071498 |
| *LINC01615* | chr6:169557257-169563019 | 0.07117 |
| *SLC20A1P3* | chr15:20172667-20173605 | 0.070809 |
| *LINC01692* | chr21:26212864-26430059 | 0.069173 |
| *AL356413.1* | chr13:86268072-86357338 | 0.068943 |
| *WT1* | chr11:32409321-32457431 | 0.066487 |
| *IGHD3-10* | chr14:106370355-106370385 | 0.064833 |
| *AC003984.1* | chr7:84161792-84213638 | 0.06417 |
| *AL512625.3* | chr9:66512823-66553911 | 0.063472 |
| *AFDN-DT* | chr6:168224556-168227389 | 0.062845 |
| *AC092445.1* | chr4:107446558-107859797 | 0.062181 |
| *ODF3B* | chr22:50968139-50971009 | 0.06038 |
| *LINC01821* | chr2:195208993-195438152 | 0.060364 |
| *GABRG3* | chr15:27216328-27787130 | 0.059934 |
| *AF212831.1* | chr21:18235195-18246011 | 0.056074 |
| *AC012038.1* | chr12:43373057-43390289 | 0.053545 |
| *HERC2P9* | chr15:28834638-28930410 | 0.053354 |
| *RPL23AP87* | chr17:81174666-81188573 | 0.051795 |
| *BAGE2* | chr21:10996026-11098980 | 0.051554 |
| *HLA-DQB1* | chr6:32627244-32636160 | 0.048963 |
| *MIR548XHG* | chr21:19933583-20132321 | 0.047336 |
| *AC093249.1* | chr16:30638297-30638499 | 0.045453 |
| *HLA-DQA1* | chr6:32595956-32614839 | 0.044936 |
| *AC105105.2* | chr18:56083997-56084860 | 0.044293 |
| *FAM86B1* | chr8:12039605-12051642 | 0.04072 |
| *RSPH10B2* | chr7:6793740-6838996 | 0.039878 |
| *GATSL2* | chr7:74807499-74867509 | 0.033843 |
| *AC022796.1* | chr11:38639814-38667906 | 0.032037 |
| *AC239800.1* | chr1:149239900-149255318 | 0.030216 |
| *AL137058.1* | chr13:53063128-53161230 | -0.02165 |
| *RPS15AP27* | chr9:110725984-110726326 | -0.0244 |
| *RNU6-756P* | chr8:125272887-125272993 | -0.03072 |
| *AC005363.2* | chr16:2021656-2021897 | -0.03184 |
| *WI2-3658N16.1* | chr1:146032648-146253110 | -0.03478 |
| *AMD1P1* | chr10:20638978-20640029 | -0.03674 |
| *SLC9A3P1* | chr10:51910363-51912047 | -0.04146 |
| *RNU6-763P* | chr2:175886942-175887047 | -0.04184 |
| *AL356968.1* | chr1:48962935-48963408 | -0.042 |
| *RNU6-929P* | chr20:55361037-55361143 | -0.04367 |
| *KRT31* | chr17:39549973-39553860 | -0.0447 |
| *RNU6-563P* | chr20:46121722-46121829 | -0.04635 |
| *AF228730.2* | chr8:6969836-6971250 | -0.04737 |
| *TRIAP1P1* | chr10:27667067-27667291 | -0.04764 |
| *AC009108.1* | chr16:86559063-86559858 | -0.04771 |
| *PRSS3P3* | chr7:141987080-141991423 | -0.04805 |
| *CGB8* | chr19:49550895-49552363 | -0.04957 |
| *AC018358.1* | chr3:42306481-42307882 | -0.05145 |
| *EEF1A1P38* | chr16:27144804-27146500 | -0.05941 |
| *AL161670.1* | chr14:64228731-64228867 | -0.05968 |
| *KRT12* | chr17:39017195-39023476 | -0.0613 |
| *KRT16P4* | chr17:18353558-18379234 | -0.06516 |
| *AC079753.1* | chr2:113601888-113601988 | -0.06636 |
| *AC012322.1* | chr16:64276512-64377813 | -0.06918 |
| *AL354897.1* | chr9:87782261-87809198 | -0.07473 |
| *COL6A3* | chr2:238232646-238323018 | -0.07975 |
| *FAM85B* | chr8:8025341-8084136 | -0.09387 |
| *HRASLS* | chr3:192958914-192995527 | -0.09855 |
| *LINC01602* | chr8:58768059-58896685 | -0.10296 |
| *ATP13A5* | chr3:192992579-193096632 | -0.10358 |
| *PKP2* | chr12:32943679-33049774 | -0.13076 |

**Table S5. Prediction using the 5hmC diagnostic model in the test set of AML patients.**

Non-CR, patients not in complete remission; CR, patients in complete remission.

|  | **AML Non-CR** | **AML CR** | **Control** |
| --- | --- | --- | --- |
| **Wd-score high** | 12 | 10 | 0 |
| **Wd-score low** | 2 | 12 | 38 |
| **Total** | 14 | 22 | 39 |
| **Sensitivity** | 85.7% | 45.5% | - |
| **Specificity** | - | - | 97.4% |

**Table S6. Multivariate Cox proportional hazards model analysis of age, sex, and wp-score in association with AML survival.** Model likelihood-ratio test *P* value is 4.00e-07.

| Variable | HR (95% CI) | *P* value |
| --- | --- | --- |
| Age | 1.00 (0.98-1.02) | .79 |
| Sex | 0.91 (0.50-1.64) | .75 |
| Wp-score | 2.33 (1.71-3.19) | 1.06e-07 |

HR, hazard ratio. CI, confidence interval.

**Table S7. Characteristics of AML cohort.**

|  | **No. of patients** |
| --- | --- |
| **Total** | 103 |
| **Sex (male/female)** | 58/45 |
| **Age (range, median)**  All  ≥60 y  <60 y | 103 (27-94, 61 Yr)  54 (60-94, 70 Yr)  49 (27-59, 49 Yr) |
| **AML classification**  De novo  Secondary  Transformed from MDS  Transformed from myelofibrosis  Transformed from CML  Transformed from CMML  tAML  World Health Organization (WHO)  AML with recurrent genetic abnormalities  AML with t(8;21)(q22;q22.1)  AML with inv(16)(p13.1q22) or t(16;16)(p13.1;q22)*^  AML with t(9;11)(p21.3;q23.3)*  AML with *BCR-ABL1*^  AML with mutated *NPM1*  AML with myelodysplasia-related changes  Therapy-related myeloid neoplasms  AML, NOS  AML without maturation  AML with maturation  Acute myelomonocytic leukemia  Acute monoblastic/monocytic leukemia  Pure erythroid leukemia  Unspecified  French-American-British (FAB)  M1  M2  M4  M5  M6  Unspecified | 88  8  2  2  1  2  3  6  3  3  9  6  2  1  3  3  4  3  60  1  4  7  8  3  80 |
| **Treatment**  No treatment  Chemotherapy  HSCT  Treatment information not available | 8  94  42  1 |
| **Cytogenetics**  Normal  Abnormal  Unknown | 43  44  16 |
| **Gene mutations**  (positive/total No. of patients tested)  *FLT3*  *NPM1*  Both *FLT3* and *NPM1*  *TET2*  *IDH1/2*  *DNMT3A*  *ASXL1*  *TP53* | 13/46  9/55  4/46  7/47  10/52  10/48  6/47  9/49 |

MDS, myelodysplastic syndrome; CML, chronic myelogenous leukemia; CMML, chronic myelomonocytic leukemia; tAML, therapy-related AML; HSCT, hematopoietic stem cell transplant; Yr, year old; * One patient with inv(16)(p13.1q22) and one patient with t(9;11)(p21.3;q23.3) also had therapy-related AML; ^ One patient with inv(16)(p13.1q22) also had *BCR-ABL1*.

**Table S8.** **Number of blood samples per AML patient in this study.** Blood from 37 patients was collected at different time points.

| Blood samples per patient | Number of patients |
| --- | --- |
| 1 | 66 |
| 2 | 22 |
| 3 | 13 |
| 4 | 1 |
| 5 | 1 |

**Table S9. Abnormal cytogenetics of AML patients.**

| **ID** | **Karyotype at diagnosis** |
| --- | --- |
| **L6** | 46,XY,t(1;15)(q25;q26)[13] |
| **L36** | 45,XY,-7,add(8)(q24),del(16)(q22)[12] |
| **L35** | 46,XY,t(9;22)(q34;q11.2),add(19)(q13.3)[4] |
| **L32** | 44,XX,add(4)(?q33),-5,add(7)(q?32),add(12)(p13),r(13),-17[14]/43,idem,-22[6] |
| **L29** | 45~47,XY,del(5)(q21q33),+6,-7,-8,t(9;22)(q34;q11.2),add(11)(pter),-15,-17x2,+4-5 mar[3]/43-45,idem,-6,-9[8]/46,XY[9] |
| **L27** | 46,XX,t(9;11)(p21.3;q23.3)[20] |
| **L25** | 46,XX,add(14)(q32)[10]/46,XX[10] |
| **L22** | 39~41,XY,t(2;12)(q33;p11.2),-4,-5,-6,-8,-13,-14,-15,-16,-17,-18,-20,-21,+mar1,+mar2,+mar3,+mar4[cp6]/37~41,XY,-4,-5,-8,-13,-14,-15,-16,-17,-18,-20,+mar1,+mar2,+mar6[cp5]/46,XY[9] |
| **BL95** | 46,XY,del(7)(q21)[16]/46,sl,del(4)(q27)[2]/46,sdl1,-del(4)(q27),add(17)(p13)[2] |
| **BL84** | 46,XY,del(20)(q11.2)[2]/48,idem,+13,+19[16]/48,idem,add(2)(q?),del(4)(q?28),+13,+19[2] |
| **BL83** | 93~49,XX,add(5)(q11.2),+8,add(13)(p11.2),+15,+mar[cp3]/46,XX[8] |
| **BL75** | 42-49,XY,i(3)(q10),der(5)t(5;17)(q11.2;q11.2),der(6)t(6;15)(q12;q11.1),-7,-13,-15,-17,+22,+1~6mar[cp20] |
| **BL66** | 46-50,XX,+1,-5,add(8)(q24.1),+add(11)(p15),+add(16)(q23),-20,-22,+add(22)(q13), +1-4mar[cp20] |
| **BL33** | 47,XY,inv(9)(p12q13),+13[9]/46,XY,inv(9)[11] |
| **BL3** | 47,XY,+8[13]/94,idemx2[2]/46,XY[5] |
| **BL29** | 46,XX,t(9;11)(p22;23)[12]/47,idem,der(9)t(9:11)[8] |
| **BL25** | 46,XX,del(7)(q22q34),inv(16)(p13.1q22)[7] |
| **BL24** | 47,XY,+8[2]/46,XY[18] |
| **BL22** | 47,XY,+6[5]/46,XY[15] |
| **BL2** | 45,X,-Y[5]/46,XY[8] |
| **BL18** | 43~47,-X,-Y,del(3)(p21),-4,add(5)(q31),dup(8)(q13q24.1),del(9)(q13q22),inv(11) |
| **BL15** | 46,XY,t(8;21)(q22;q22)[16]/46,XY[4] |
| **BL14** | 47,XX,t(10;15)(q22;q15),inv(16)(p13.1q22),+22[19]/46,XX[1] |
| **BL13** | 46,XY,t(8;21)(q22;q22)[4] |
| **BL117** | 46,XX,inv(16)(p13q22)[20] |
| **BL115** | 46,XY,t(16;16)(p13;q22)[18]/46,XY[2] |
| **BL104** | 46,XX,der(7)add(7)(p11)t(7:11)(q32;q13)[18]/46,XX[2] |
| **BL1** | 46,XY,t(3;19)(p21;p13.3)[cp6]/46,XY[14] |
| **B3L44** | 46,XY,inv(16)(p13q22),t(9;22)(q34;q11.2)[20] |
| **B3L42** | 42,XX,del(5)(q12q33),der(13;22)(q10;q10),-15,der(17)t(17;17)(p13;q15),-18,t(19;21)(q13.3;q22),-22x2[20] |
| **B3L28** | 45,XY,-7[13]/45,idem,t(2;6)(q31;q23),t(15;19)(q21;q13.4)[3]/46,XY[4] |
| **B3L17** | 45,XY,der(13;14)(q10;q10)[20] |
| **4B-50** | 46,XX,t(9;11)(p21.3;23.3)[12]/47,idem,der(9)t(9:11)[8] |
| **4B-5** | 47,XX,+8[15]/47,idem,del(7)(q22q34)[3]/46,XX[2] |
| **4B-49** | 46,XX,del(1)(p13),add(5)(q13),der(7)t(1;7)(p13;q11.2),der(17)(17pter->17q21::?::5q13->5qter)[8]/76<3n>,XXX,del(1)(p13),add(5)(q13)x2,+6,+8,+14,+17,der(17)(17pter->17q21::?::5q13->5qter)x2,+18,+19,+21[5]/46,XX[7] |
| **4B-41** | 46,XX,t(1;13)(p12;p13),der(3)t(3;21)(q25,q21),der(21)t(3;21)(q25;q22)[12]/46,i dem,del(7)(q22q34)[5]/46,XX[3] |
| **4B-36** | 47,XY,+8[5]/47,idem,add(8)(q21)[11]/46,XY[4] |
| **4B-26** | 46,XX,t(3;5)(q24;q34)[20] |
| **4B-24** | 46,XY,inv(16)(p13.1q22)[20] |
| **4B-22** | 45,XX,-7[18]/46,XX[2] |
| **4B-20** | 46,XX,t(8;21)(q22;q22.1)[2]/45,idem,-X[13]/46,XX[5] |
| **4B-19** | 46,XX,inv(16)(p13.1q22)[7]/47,idem,+22[10]/46,XX[3] |
| **4B-16** | 49,XY,+add(1)(p13),+8,+9,+16[9]/46,XY[11] |
| **4B-1** | 46,XX,t(1;3)[20] |

**
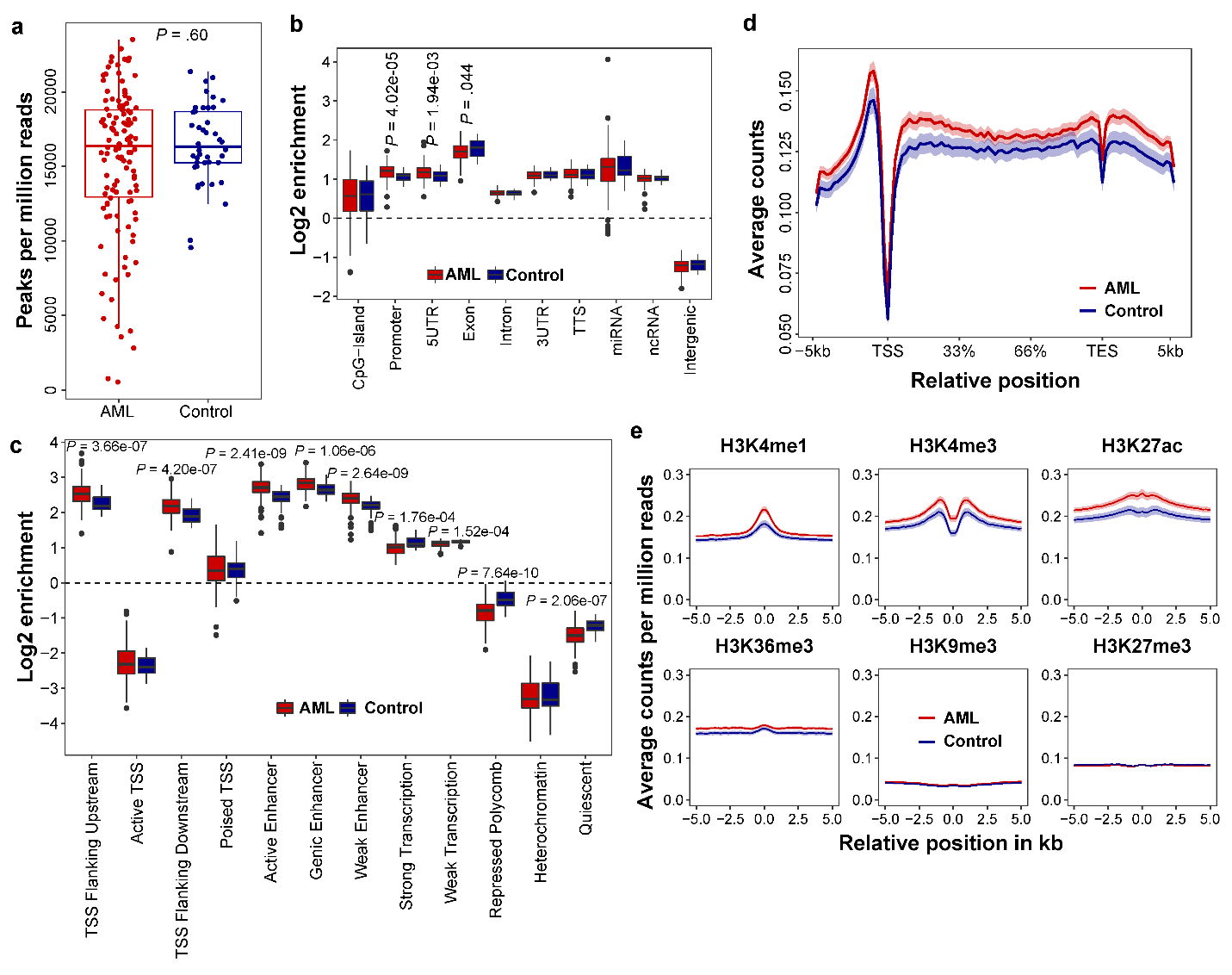
**

**Figure S1. Genome-wide distribution of plasma cell-free DNA 5-hydroxymethylcytosine (5hmC) in acute myeloid leukemia (AML) and control samples.** (a) Genome-wide enrichment of 5hmC in 122 AML and 42 control samples. Each dot represents normalized 5hmC peak numbers per million reads from each sample. (b) Boxplot of 5hmC peak enrichment analysis in AML and control samples. Center line represents median, bounds of box represent 25th and 75th percentiles, and whiskers are Tukey whiskers. *P* values < .05 are indicated. (c) Enrichment of 5hmC peaks in cis-regulatory elements. TSS, transcription start sites. *P* values from two-sided Wilcoxon rank sum test. Center line represents median, bounds of box represent 25th and 75th percentiles, and whiskers are Tukey whiskers. (d) Metagene profile of normalized 5hmC reads in gene body regions in AML and control samples. (e) Metagene profiles of normalized 5hmC reads in genomic regions associated with histone marks. Shaded regions in (d) and (e) are 95% confidence interval.

**
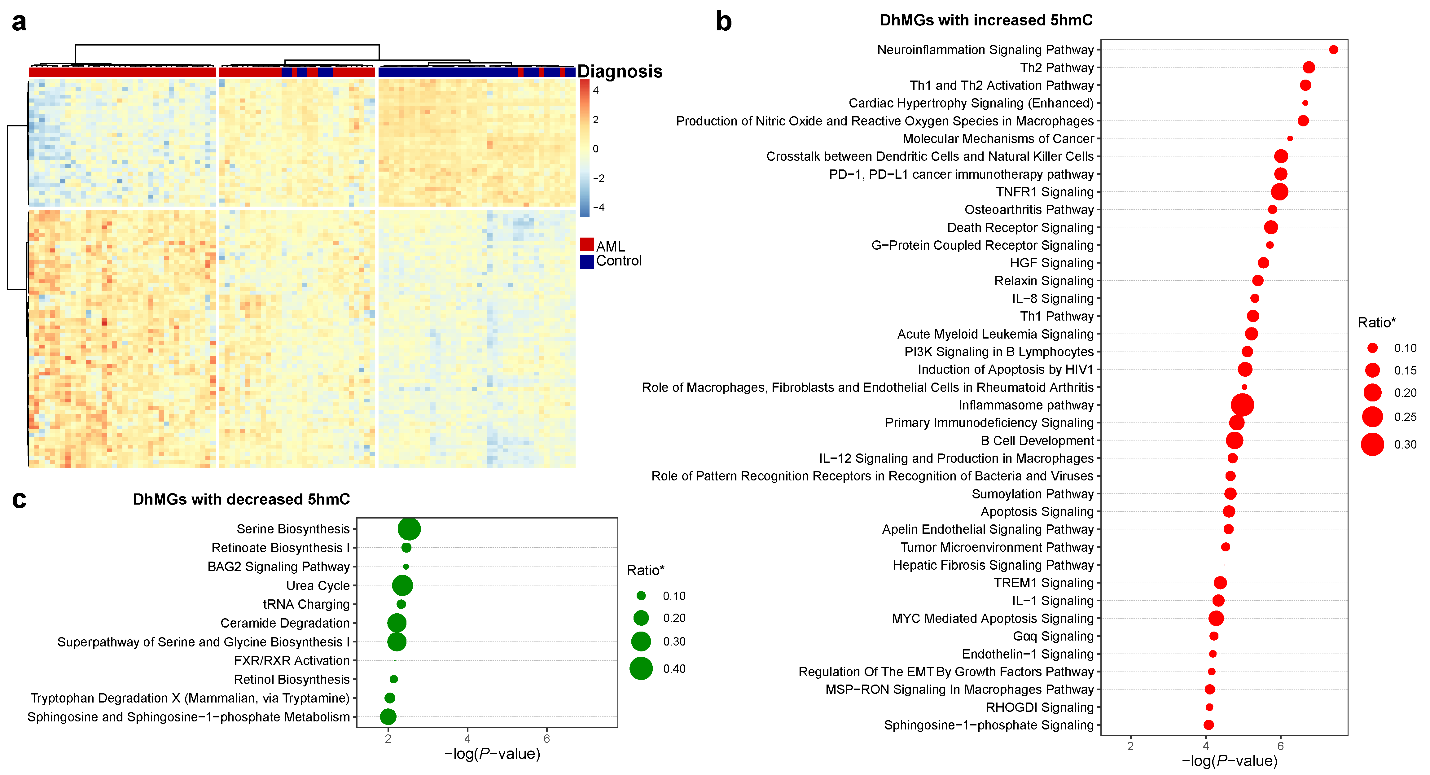
**

**Figure S2. Differentially hydroxymethylated genes (DhMGs).** (a) Unsupervised hierarchical clustering of top significant 100 DhMGs in AML patient samples at registration time points and controls. Each row represents one DhMG; each column represents one sample. (b) Pathway enrichment analysis of DhMGs with increased 5hmC and expression levels using Ingenuity Pathway Analysis (IPA) (*P* < 1e-4). (c) Pathway enrichment analysis of DhMGs with decreased 5hmC and expression levels using IPA (*P* < 1e-2).


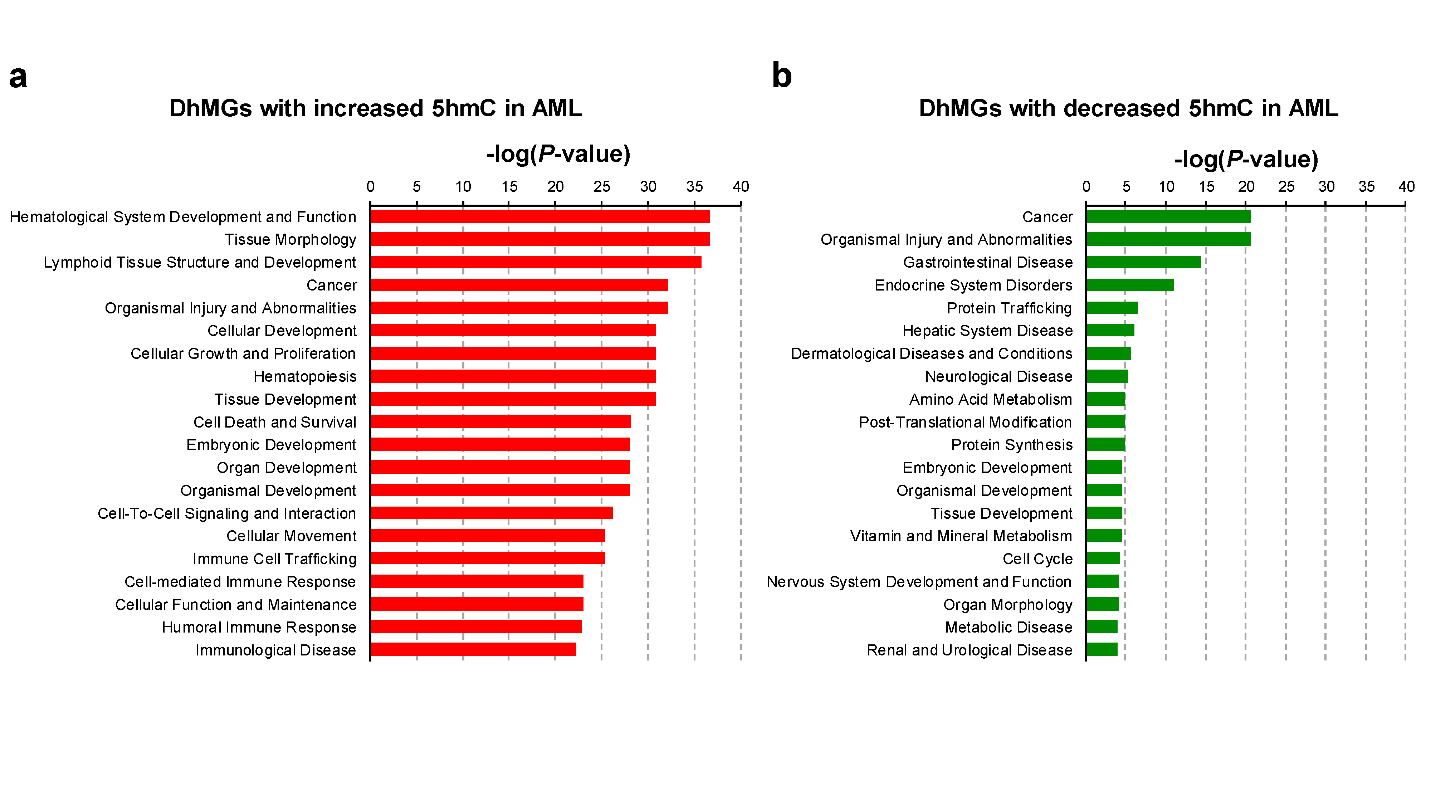


**Figure S3. Pathway enrichment analysis of DhMGs.** (a) Disease and function enrichment of DhMGs with increased 5hmC levels using Ingenuity Pathway Analysis (IPA). (b) Disease and function enrichment of DhMGs with decreased 5hmC levels using IPA.


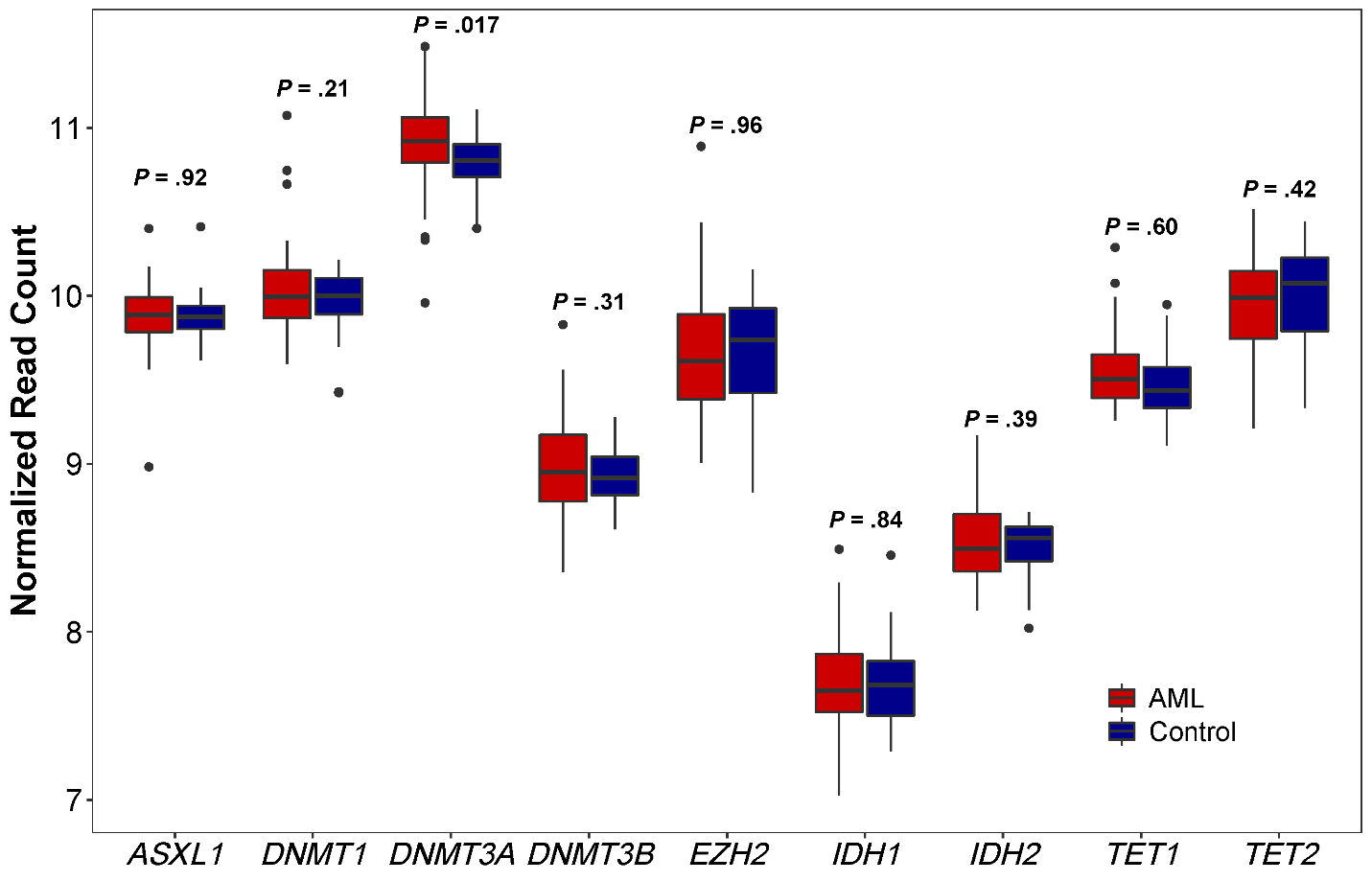


**Figure S4.** **Boxplot of 5hmC enrichment in genes associated with epigenetic modifications.** 5hmC enrichment of genes associated with epigenetic modifications in 122 AML and 42 control samples. Adjusted *P* values are from DESeq2. Center line represents median, bounds of box represent 25th and 75th percentiles, and whiskers are Tukey whiskers.

122 specimens from 76 patients before 2020

76 specimens collected at registration

46 specimens collected at different time points

62 specimens with overt leukemia

Diagnostic model training set - 37 specimens

Diagnostic model validation set - 25 specimens

Predictive model training set - 50 specimens

Predictive model validation set - 26 specimens

36 specimens from 27 patients in 2020 and 2021

158 specimens from 103 patients

Diagnostic model test set - 36 specimens

Identify differentially hydroxymethylated genes in AML

**Figure S5.** **Flow chart of study design of AML specimens for 5hmC diagnostic and predictive models.**


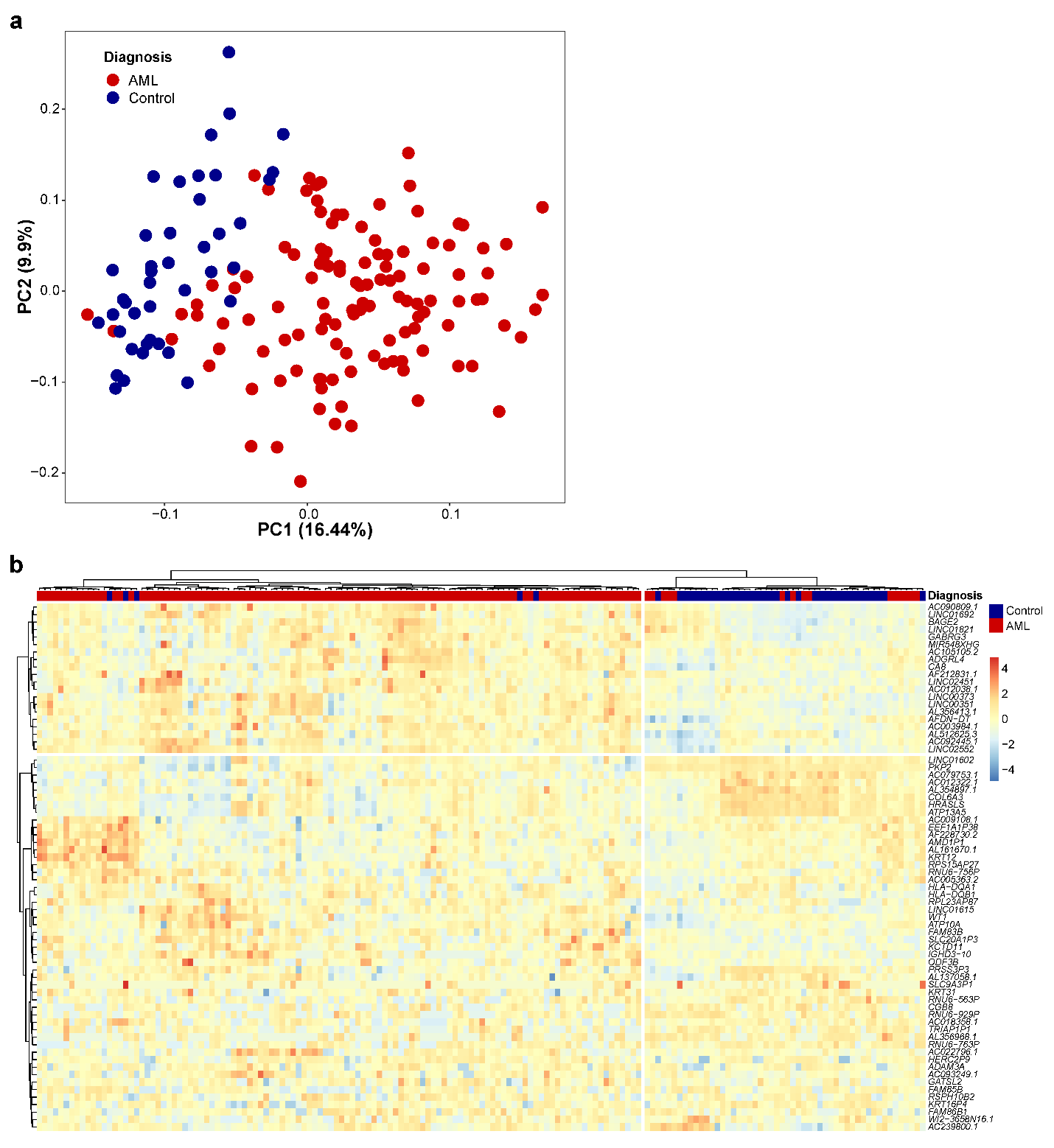


**Figure S6. A 5hmC signature of 70 genes differentiates AML patients from controls.** (a) Principal component analysis (PCA) in 122 AML samples collected before 2020 and 42 controls using normalized read counts from the 5hmC signature of 70 genes. (b) Unsupervised hierarchical clustering of the 5hmC signature of 70 genes in 122 AML samples collected before 2020 and 42 controls.

**
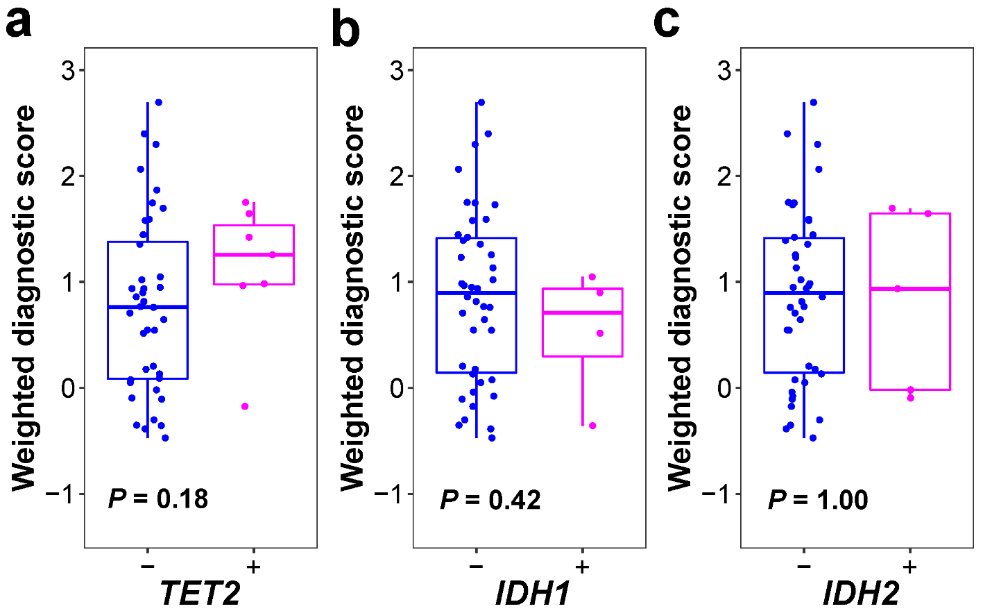
**

**Figure S7. 5hmC diagnostic signature in patients with or without a mutation in DNA demethylation related genes.** (a) Boxplot of weighted-diagnostic scores (wd-scores) in patients with (n = 7) or without (n = 40) a mutation in *TET2*. -, without a mutation in *TET2*; +, with a mutation in *TET2*. (b) Boxplot of wd-scores in patients with (n = 4) or without (n = 42) a mutation in *IDH1.* -, without a mutation in *IDH1*; +, with a mutation in *IDH1*. (c) Boxplot of wd-scores in patients with (n = 5) or without (n = 42) a mutation in *IDH2*. -, without a mutation in *IDH2*; +, with a mutation in *IDH2*.


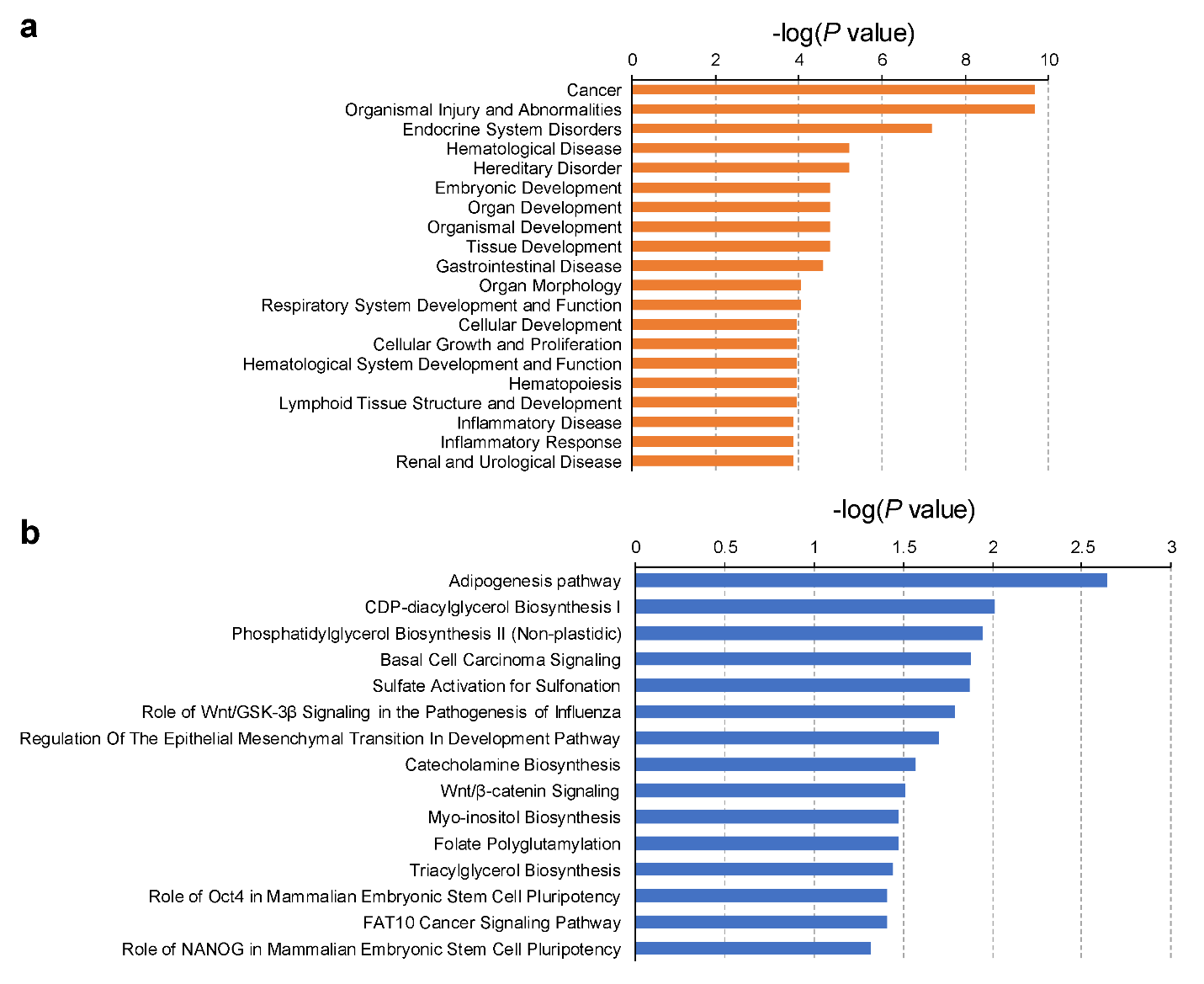


**Figure S8. Pathway enrichment analysis of 279 survival-related DhMGs using Ingenuity Pathway Analysis.** (a) Disease and function categories enriched in 279 survival-related DhMGs. Top 20 disease and function categories are shown. (b) Pathways enriched in 279 survival-related DhMGs. *P* < .05.


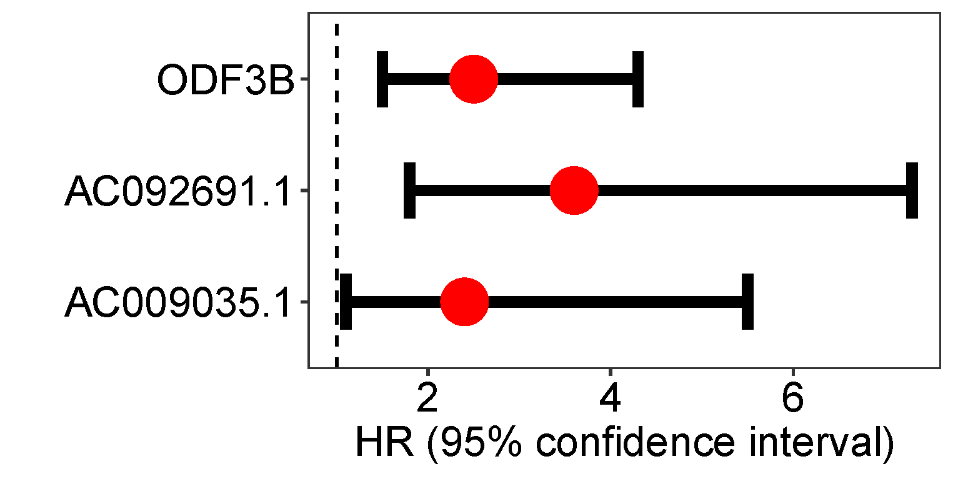


**Figure S9. A 5hmC signature of three genes significantly associated with overall survival.** HR, hazard ratio.


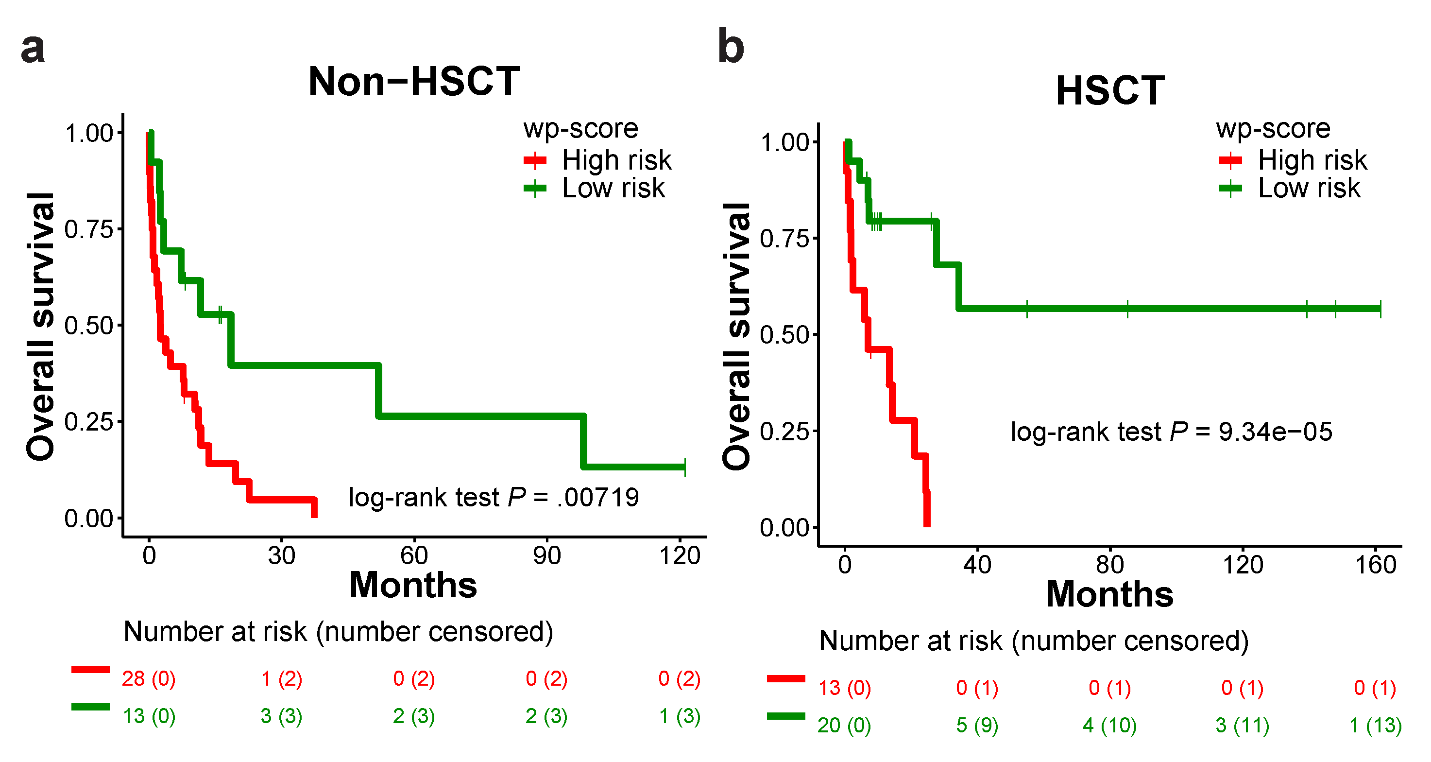


**Figure S10. Overall survival analysis of AML patients.** (a) Overall survival analysis of AML patients that did not receive hematopoietic stem cell transplant (HSCT) (n = 41). (b) Overall survival analysis of AML patients that received HSCT (n = 33). “|” represents censored data.

**
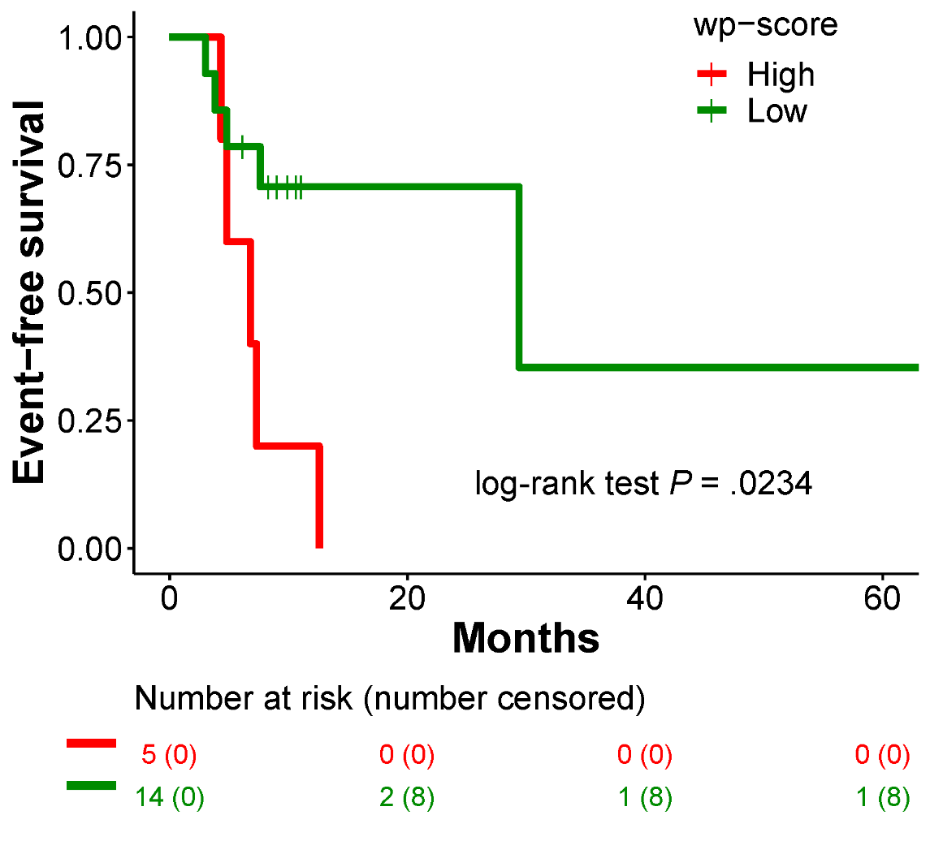
**

**Figure S11. The survival-related 5hmC signature is associated with event-free survival in AML patients.** Kaplan-Meier survival analysis of AML patients based on wp-score. “|” represents censored data.


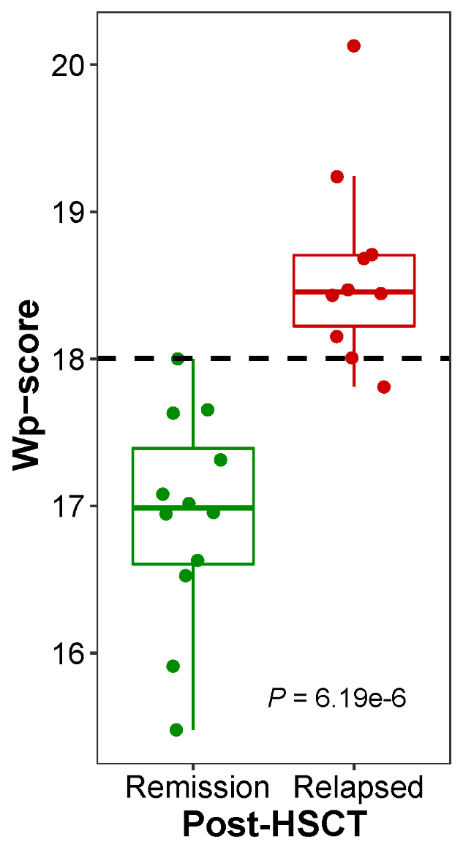


**Figure S12. The** **survival-related 5hmC signature predicts leukemia burden in post-HSCT patients.**  Boxplot of wp-scores in post-HSCT patients who remain in complete remission (n = 12) or who later became relapsed (n = 10).


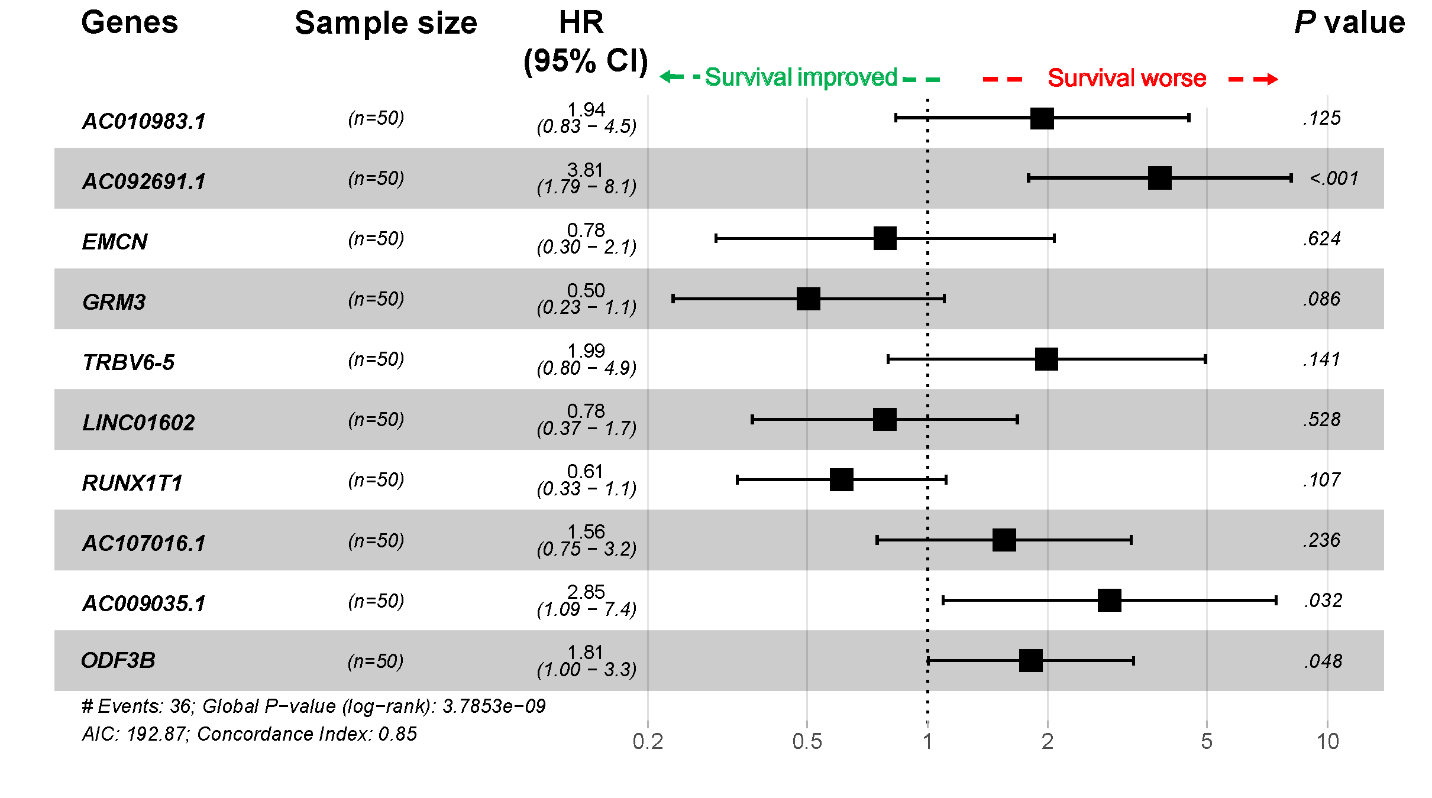


**Figure S13.** **Candidate genes for final survival-related 5hmC signature.** Ten genes were selected using elastic net model by cross-validation in at least 95% iterations in 100 sampling events. HR, hazard ratio. CI, confidence interval.
